# Optimization of family sizes in sets of crosses with a greedy allocation strategy based on automatic differentiation

**DOI:** 10.3389/fpls.2026.1727383

**Published:** 2026-04-30

**Authors:** Uche Joshua Okoye, Matthias Frisch, Eva Herzog

**Affiliations:** Institute of Agronomy and Plant Breeding II, Justus Liebig University, Giessen, Germany

**Keywords:** automatic differentiation, cross planning, greedy optimization, segregation variance, truncation selection

## Abstract

Line breeding programs of autogamous crops like barley and wheat are characterized by multiple crosses with high variability in cross means and segregation variances. From theory of order statistics, we derived formulas for the expected selection differential, the response to selection, and the expected genetic value of the total selected fraction from all crosses. With these target functions, we developed a greedy optimization algorithm based on automatic differentiation to maximize the expected genetic value in the next breeding cycle by optimizing the allocation of a fixed total number of individuals to the various crosses. We show mathematically that the optimization algorithm yields the global optimum of progeny allocation for a fixed set of crosses under the constraint of fixed total population size and the given size of the selected fraction per cross. We tested the optimization algorithm with an experimental dataset from barley resistance breeding and with 60 simulated datasets characterized by different ratios of the variance of the segregation standard deviations and the variance of the cross means. For every scenario, we investigated population sizes of 400 and 1,200 individuals and heritabilities of 0.7 and 0.9. Optimized family sizes consistently outperformed constant family sizes, particularly in scenarios with high variability in segregation variances and a large ratio of the variance of the segregation standard deviations and the variance of the cross means. The relationship between the segregation variances and the optimal family sizes was approximately linear. The results indicate that the optimal allocation of individuals to crosses is dataset-specific and cannot be derived from a single heuristic criterion. The study highlights the importance of considering segregation variances in optimizing family sizes and offers user-friendly R code for improving the efficiency of line breeding programs.

## 1 Introduction

Line breeding programs of autogamous crops like barley and wheat rely on multiple crosses of homozygous parent lines. From the offspring of these crosses, a certain number of inbred lines is derived that form the selection candidates for the next breeding cycle. By directional selection, breeders improve the mean genetic value of the population while maintaining a sufficient level of diversity for long-term selection gain. Important factors for enhancing the response to selection in such breeding programs are the selection of cross parents with desired characteristics, optimized between-cross and within-cross selection, and optimal resource allocation in terms of number of individuals generated and selected per cross.

We consider the establishment of a new breeding pool with the aim of generating high-yielding selection candidates with additional desirable agronomic properties. The parent lines of crosses in such newly established breeding pools usually consist of genetically diverse donor and elite genotypes. Crosses of these donor and elite lines in the initial phases of pool establishment are deliberately planned by breeders by suitable methods of parent selection and between-cross selection to enhance and maintain genetic diversity in the elite material and to introgress traits like resistances into elite backgrounds. Typical use cases could be the founding of new resistance breeding pools or new heterotic pools for hybrid breeding. In order to maintain the diversity generated by the deliberately planned crosses *i* = 1, 2, *…*, *k*, all crosses should contribute as evenly as possible to the following cycle.

Once parent selection and between-cross selection are finalized, the next step is to improve selection gain from within-cross selection under budget restrictions that limit both the total number of individuals that can be generated from the planned crosses as well as the number of individuals that can be selected for further testing. Under the scenario of within-cross selection with a fixed set of pre-selected crosses from which a constant number of individuals *s_i_* per cross is to be selected for their yield potential, the most important factor for maximization of the genetic value of the selected fraction is an optimal allocation of the limited total number *N* of individuals that can be generated and tested to the *k* crosses.

While extensive research has been conducted on optimal selection and contribution of cross parents and between-cross selection (Schnell and Utz, 1975; Meuwissen and Sonesson, 1998; Zhong and Jannink, 2007; Daetwyler et al., 2015; Akdemir and Sánchez, 2016; Goiffon et al., 2017; Lehermeier et al., 2017; Gorjanc et al., 2018; Müller et al., 2018; Allier et al., 2019; Wellmann, 2019; Bijma et al., 2020; Sakurai et al., 2024; Kinoshita et al., 2025; Peixoto et al., 2025), the optimal allocation of family sizes *n_i_* for within-cross selection under the restriction of a fixed total population size *N* and a fixed number of selected individuals *s_i_* per cross has received less attention.

A wide range of studies have addressed progeny allocation in cross planning (Bernardo, 2003; Hunter and McClosky, 2016; Moeinizade et al., 2021; Hassanpour et al., 2023; Danguy des Déserts et al., 2023; Hamazaki and Iwata, 2024). These studies were based on replicated or multi-cycle simulation pipelines that might be computationally expensive to run, use complex black-box optimization algorithms, and, in most cases, do not separate between-cross selection from the problem of progeny allocation for within-cross selection. To our knowledge, no explicit optimal solution exists that tackles the optimization of family sizes *n_i_* for a fixed set of *k* pre-selected crosses for within-cross selection. A fast, user-friendly implementation of an optimization algorithm for this problem would support breeders in both short-term and long-term breeding decisions without need to run time-consuming simulations.

Inbred offspring from donor × elite crosses often display a low mean performance but a high segregation variance for yield, while inbred offspring from elite × elite crosses can be expected to display a high mean performance and low segregation variance. This can be explained by different levels of relatedness and genetic diversity of the cross parents. Thus, new breeding pools are often characterized by a high variation of the segregation variances.

The formula *R* = *iHσ*^∗^ (cf. Falconer and Mackay, 1996, p. 192), where *i* is the standardized selection differential, *H* is the square root of the heritability, and *σ*^∗^ is the segregation standard deviation, underlines that the cross-specific response to selection is a function of the segregation variance. Applying the approximation for the selection differential by Burrows (1972) for finite population sizes, it can be shown that crosses with high segregation variance have a greater potential for improvement of *R* with increasing family size *n_i_* than crosses with low segregation variance. It has therefore been suggested to allocate family sizes for a fixed set of crosses proportionally to the segregation standard deviations of the crosses (Oget-Ebrad et al., 2024), or proportional to the genetic diversity of the parents (Moeinizade et al., 2019). These are very sensible heuristic allocations, but no explicit optimal solutions. The nature of the relationship between optimal *n_i_* and the family-specific segregation variances at different levels of total population size *N* has not yet been investigated in detail.

The maximization of the total response to selection as a function of the family sizes of the considered crosses requires a target function that yields the total expectation of the response to selection over all crosses, and can consider both finite family sizes and finite numbers of selected individuals. In the present study, we derive a solution for this total response to selection based on theory of order statistics (Harter, 1961; David and Nagaraja, 2003). This has previously been suggested by Burrows for single crosses (Burrows, 1972) and made use of by Müller et al. (2018), but has not yet been derived and implemented for a set of multiple crosses.

Applying the approximation by Burrows and assuming a constant within-cross variance, Huehn (2005) showed that the total response to selection *R* is continuously increasing with increasing total population size *N*. However, the approach of Huehn (2005) only considers constant within-cross variance and cannot handle variable cross-specific segregation variances.

A promising optimization approach could be to gradually assign individuals to the crosses where the marginal response to selection is largest, thus following the steepest gradient of the target function. For this purpose, the gradients of the cross-specific curves for the response to selection (that directly depend on the segregation variances of crosses) can be computed. The free open-source library Torch provides functionality for automatic differentiation and gradient ascent for this purpose and has user-friendly interfaces to Python and R (Paszke et al., 2017, 2019; Falbel and Luraschi, 2025).

The goal of this study was to develop methodology for optimal progeny allocation with the intent to maximize the total response to selection from multiple crosses in the framework of within-cross selection. We consider a set of *k* pre-selected crosses with varying means and segregation variances and optimize progeny allocation under the constraints of finite total population size *N* and constant size of the cross-specific selected fraction. In detail, the objectives were to (1) apply theory of order statistics to derive formulas for the expectation, the response to selection, and the genetic value of the total selected fraction from multiple crosses with finite variable family sizes *n_i_* and variable means and segregation variances; (2) develop an efficient greedy optimization algorithm based on the principle of automatic differentiation for maximizing the expected genetic value of the total selected fraction by optimizing the allocation of *N* = $\sum n_{i}$ individuals to the *k* crosses; (3) compare the effect of optimized family sizes *n_i_* on selection gain to scenarios with constant family sizes for different total population sizes *N* and different number of selected individuals *s_i_*; and (4) investigate the relationships between the optimal *n_i_*, the segregation variances $\sigma_{i}^{*2}$, and the cross means *µ_i_*.

## 2 Materials and methods

### 2.1 Order statistics for estimation of the expectation of the selected fraction

We consider a base population consisting of *k* families of inbred lines of size *n_i_* (*i* = 1, …, *k*). For each family, the mean of a trait *µ_i_* and the genotypic segregation variance $\sigma_{i}^{*2}$ can be estimated from the marker genotypes of the parental lines using the approach suggested by Osthushenrich et al. (2017).

Selection is carried out for phenotypic values obtained from a field trial with heritability *H*^2^. Hence, the variance of the phenotypic values is $\sigma_{i}^{2}$ = $\sigma_{i}^{*2}$/*H*^2^ and the probability distribution has the density *f*(*x_i_*) = *φ* (*x_i_*, *µ_i_*, *σ_i_*), where *φ* is the density function of the normal distribution with mean *µ_i_* and standard deviation *σ_i_*.

Let *X* denote a random variable that describes the genotypic values in the base population. The distribution of *X* is a finite mixture distribution of *X*_1_, …, *X_k_* with probability density function

(1) $$f(x)=\sum_{i=1}^{k}w_{i}f(x_{i})$$

with weights

(2) $$w_{i}=\frac{n_{i}}{N}\text{ and }N=\sum_{i=1}^{k}n_{i}$$

where *n_i_* is the size of the *i*th family and *N* is the size of the base population consisting of the *k* families. The expectation of the base population is

(3) $$\text{E}(X)=\sum_{i=1}^{k}w_{i}\text{E}(X_{i})=\sum_{i=1}^{k}w_{i}\mu_{i}$$

We now consider selection of the *s_i_* best lines from the *n_i_* lines of family *i*. Let the random variable *Y_i_* describe the selected fraction of family *i*. *Y_i_* is a finite mixture of the *s_i_* greatest order statistics *X_i_*(*n_i_*−*s_i_* + 1), …, *X_i_*(*n_i_*−1), *X_i_*(*n_i_*) of *X_i_*. The expectation of the *r*th-order statistic (*r* = *n_i_* − *s_i_* + 1, …, *n_i_*) of a normal distribution is defined (cf. David and Nagaraja, 2003, pp. 33–34)

(4) $$\text{E}(X_{i(r)})=\int_{-\infty }^{\infty }x_{i}f_{\left(r\right)}(x_{i})dx_{i}$$

The expectation for the selected fraction of family *i* is

(5) $$\text{E}\left(Y_{i}\right)=\frac{1}{s_{i}}\sum_{r=n_{i}-s_{i}+1}^{n_{i}}\text{E}\left(X_{i\left(r\right)}\right)$$

The expectation of the selected fraction *Y* of the base population *X* is a mixture of the expectations E(*Y_i_*) of the selected fractions *Y_i_* of the individual families; therefore,

(6) $$\text{E}\left(Y\right)=\sum_{i=1}^{k}v_{i}\text{E}\left(Y_{i}\right)$$

where the weights are

(7) $$v_{i}=\frac{s_{i}}{N_{s}}\text{ and }N_{s}=\sum_{i=1}^{k}s_{i}$$

The expected selection differential is then (cf. Falconer and Mackay, 1996, p. 188) *S* = E(*Y*) − E(*X*), and the selection gain is *R* = *H*^2^*S*.

### 2.2 Calculation of the expected genetic value E(*G*)

We assumed that a constant number of individuals *s_i_* is selected from each family, meaning that all the families are represented in the following generation in equal proportion, the proportion being $v_{i}=s_{i}/\sum s_{i}=s_{i}/N_{s}=1/k$. As a criterion for evaluating the optimization of the family sizes *n_i_*, the expected genetic value for each family in the following generation was calculated by E(*G_i_*) = *µ_i_*+ *R_i_* and the expected genetic value of the total selected fraction was calculated as the unweighted mean of the family-specific E(*G_i_*):

(8) $$\text{E}(G)=\frac{1}{k}\sum \text{E}(G_{i})$$

**Figure 1:**
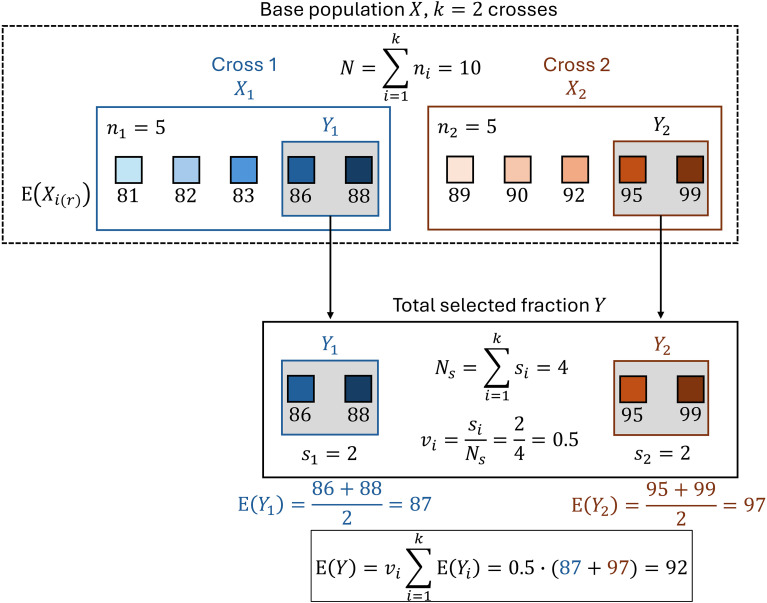
Example for the calculation of E(*Y*) from *k* = 2 crosses with constant family sizes *n_i_* = 5 and constant number of selected individuals *s_i_* = 2. The total base population *X* (dotted box) consists of the *N* = $\sum n_{i}$ = 10 individuals from the two crosses (blue and brown squares). For each cross, expectations for all order statistics $\text{E}(X_{i(r)})$ can be predicted. Their values are the numbers shown under the blue and brown squares representing the individuals. The top *s_i_* = 2 order statistics of each cross make up the cross-specific selected fraction *Y_i_* (filled gray boxes) with expectation E(*Y_i_*). The total number *N_s_* = $\sum s_{i}$ = 4 of selected individuals from the two crosses make up the total selected fraction *Y*. The expectation of the total selected fraction E(*Y*) is calculated as the weighted mean of E(*Y*_1_) and E(*Y*_2_).

To make the formulas more accessible to readers, we included an illustrative figure with a toy example for the calculation of E(*Y*) from two crosses with constant family size *n_i_* = 5 and constant number of selected individuals *s_i_* = 2 in **Figure 1**. A glossary of the variables and parameters from Equations 1 to 8 can be found in **Table 1**.

**Table 1:** List of the most frequently used mathematical symbols with explanations.

Symbol	Explanation
*µ*	Mean of the base population *X*
*µ_i_*	Mean of cross *i*
$\sigma_{i}^{*2}$	Cross-specific segregation variance
$\sigma_{i}^{2}$	Cross-specific phenotypic variance
*k*	Number of crosses
*n_i_*	Cross-specific family size of cross *i*
*N*	Total population size of base population *X*, *N* = $\sum n_{i}$
*s_i_*	Number of selected individuals from cross *i*
*N_s_*	Size of the total selected fraction *Y*, *N_s_* = $\sum s_{i}$
E(*Y_i_*)	Expectation of the cross-specific selected fraction
E(*Y*)	Expectation of the total selected fraction
E(*G_i_*)	Cross-specific expected genetic value
E(*G*)	Total expected genetic value

### 2.3 Optimization of *n_i_*

The numerical integration for calculating the expectation of order statistics and the algorithm for optimization of the family sizes *n_i_* were implemented with the R package Torch version 0.14.2 (Falbel and Luraschi, 2025) running in R 4.5.1 (R Core Team, 2025). The algorithm is an analytical optimization procedure relying on Torch’s efficient functionality for automatic differentiation. In the first step, *s_i_* individuals were assigned to each of the *k* = 20 crosses. As target function for the optimization, we considered the total E(*G*) as a function of the family sizes *n_i_*. The goal was to maximize the marginal gain in E(*G*) with every additionally assigned individual. As E(*G*) is the unweighted mean of the final values of the E(*G_i_*) curves, this can be achieved by stepwise assignment of single individuals to the family with the current largest gain in E(*G_i_*).

For this purpose, the gradient of all *k* = 20 E(*G_i_*) curves was calculated with Torch’s automatic differentiation function autograd. In every iteration of the algorithm, the next individual was always assigned to the cross with the largest gradient with the current assignment of *n_i_*. This procedure was repeated until all *N* − $\sum s_{i}$ remaining individuals were allocated. R code containing both the code for the calculation of E(*G_i_*) and the optimization of the *n_i_* with a stand-alone running example scenario for the experimental dataset is provided in the **Supplementary Materials**.

In more detail, *n_i_* are treated as continuous in the automatic differentiation, even though in reality, only integer values are feasible. The factorial (*n_i_*!) and the binomial coefficient $\left(\begin{array}{c} n_{i}-1\\ r-1 \end{array}\right)$ are calculated via gamma-function extensions of factorials. Instead of using a finite-difference scheme for calculating marginal gains, a closed-form derivative of the continuous expression is used. The partial derivative $\partial \text{E}(G_{i})/\partial n_{i}$ is computed by chain rule. In the iterations of the optimization loop, the gradients are treated as the marginal gains. The next individual is always allocated to the cross with the largest $\partial \text{E}(G_{i})/\partial n_{i}$. The gradient will then be updated for this cross. This procedure is repeated until all individuals *N* are allocated to crosses. As the gradients are always evaluated at integer points, the successively computed gradients reflect the discrete marginal gains and should be numerically identical to the marginal gains of a finite-difference approach. The procedure is therefore numerically stable.

Pseudocode for the optimization algorithm that should be executable in any language that allows for automatic differentiation of functions involving gamma functions can be found in **Appendix 1**.

### 2.4 Experimental dataset

To illustrate the effect of different allocations of family sizes *n_i_* on the selection gain E(*G*), we used a barley dataset that was evaluated for the accuracy of the prediction of cross means and segregation variances in a previous study (Osthushenrich et al., 2018). The data were originally generated for a resistance breeding experiment that was conducted in cooperation with commercial breeding companies. As an investigated trait, we considered yield in decitons per hectare (dt/ha). The five registered six-row barley varieties JEN, MER, OTT, ETI, and QUA were crossed with the four resistance donor lines 146, D33, D37, and ANT, resulting in *k* = 20 crosses from which doubled haploid (DH) lines were derived. For the *k* = 20 DH families, cross means *µ_i_* and segregation variances $\sigma_{i}^{*2}$ were estimated with the methods described in Osthushenrich et al. (2017). We treated these parameters as given “true” values for illustrating the principle of our optimization algorithm. The donor line ANT is also a registered variety. Thus, the dataset contains both elite × elite and donor × elite crosses and is characterized by a comparatively high variation of both the estimated cross means *µ_i_* and the estimated segregation standard deviations $\sigma_{i}^{*}$. The pre-estimated cross means and segregation variances from the study of Osthushenrich et al. (2018) that we used for illustrating the use of the newly derived formulas and algorithm are shown in **Table 2**. The variance of the segregation standard deviations $\sigma_{i}^{*}$ was approximately 0.05 times the variance of the cross means *µ_i_*. The heritability for yield was *H*^2^ = 0.83 in an unbalanced yield trial of the DH lines (Terraillon et al., 2022). We investigated similar values of *H*^2^ = 0.7 and 0.9 to illustrate the effect of different levels of heritability.

**Table 2:** Predicted means *µ_i_* and segregation variances $\sigma_{i}^{*2}$ for yield in (dt/ha) of *k* = 20 crosses between 9 barley lines.

	Parent 2			
Parent 1	146	ANT	D33	D37
	${µ}_{i}\sigma_{i}^{*2}$			
ETI	82.85, 8.73	94.23, 1.46	87.10, 5.23	83.53, 11.91
JEN	85.88, 11.65	97.26, 1.04	90.13, 7.32	86.56, 15.17
MER	85.08, 12.12	96.45, 1.20	89.33, 6.36	85.75, 12.75
OTT	82.95, 9.83	94.33, 0.96	87.20, 3.68	83.63, 10.06
QUA	85.93, 11.74	97.31, 1.05	90.18, 7.37	86.61, 15.20

### 2.5 Simulated datasets

In addition to the experimental dataset, we sampled cross means *µ_i_* and segregation standard deviations $\sigma_{i}^{*}$ for 60 random datasets of *k* = 20 crosses from normal distributions.

As means for random sampling of the cross means *µ_i_*, we used the grand mean and the variance of the cross means of the experimental dataset.

For sampling the segregation standard deviations $\sigma_{i}^{*}$, the variance of segregation standard deviations was scaled to be a multiple of the variance of the cross means of the experimental dataset: $\text{Var}{\left(\sigma_{i}^{*}\right)}_{\text{sim}}=c\text{Var}{\left(\mu_{i}\right)}_{\text{exp}}$. The scaling factor took values of *c* = 0.025, 0.05, 0.1, 0.5, 1, and 2. To achieve this, we set a lower boundary for the segregation standard deviations of 1 that corresponded to the lowest value observed in the experimental dataset and calculated the mean for sampling the segregation standard deviations as $1+2\sqrt{c\text{Var}{\left(\mu_{i}\right)}_{\text{exp}}}$. It can thus be expected that 95% of the sampled segregation standard deviations should fall into an interval $\left[1;1+4\sqrt{c\text{Var}{\left(\mu_{i}\right)}_{\text{exp}}}\right]$. We repeated the sampling procedure for each dataset until the deviation of the actual $\text{Var}{\left(\sigma_{i}^{*}\right)}_{\text{sim}}$ from the desired value was *<* 0.01.

### 2.6 Investigated scenarios

For both the experimental barley dataset and the 60 simulated datasets, we investigated $s_{i}=1,\;2,\;3,\;4,\;5,\;10$, $H^{2}=0.7,\;0.9$ and N=400, 1,200. For each combination of these values, we compared E(G) with optimized variable $n_{i}$ allocated with the gradient ascent algorithm to E(G) with constant $n_{i}=N/k$.

### 2.7 Sensitivity test of the optimization to variance prediction error

To test the robustness of our optimization algorithm against variance prediction error, we investigated three different levels of random prediction error for the segregation variances $\sigma_{i}^{*2}$ of the experimental dataset: 5%, 10%, and 25% of mean absolute variance error (MAVE). To obtain the MAVE values for all *k* = 20 crosses, we multiplied the segregation variances $\sigma_{i}^{*2}$ with the percentages listed above: MAVE_5%_ = 0.05 · $\sigma_{i}^{*2}$, MAVE_10%_ = 0.1 $\sigma_{i}^{*2}$, MAVE_25%_ = 0.25 $\sigma_{i}^{*2}$.

The random prediction errors were sampled from a normal distribution with μ=0 and $\sigma =\text{MAVE}\sqrt{\pi /2}$. The sampled prediction errors were added to the “true” *σ_i_*^∗2^ of the experimental dataset to calculate “noisy” variance estimates with prediction error. We used the noisy variances to optimize *n_i_* with our algorithm. We then predicted E(*G*) with these suboptimal variable *n_i_* and the “true” parameters of the experimental dataset.

We investigated combinations of *N* = 400, 1,200, *s_i_* = 1, 2, 3, 4, 5, 10, and *H*^2^ = 0.7, 0.9. Each scenario was replicated 100 times. As a measure of the loss in “true” selection gain that is incurred by using suboptimal variable *n_i_*, we calculated the difference Δ_var_= E(*G*)_optimal_ − E(*G*)_suboptimal_ for all scenarios and replications. We averaged the Δ_var_ over replications and also calculated the 5th and 95th percentiles. To check if suboptimal *n_i_* ever resulted in E(*G*) that were worse than E(*G*) with constant *n_i_*, we also evaluated the difference Δ_const_ = E(*G*)_const_ − E(*G*)_suboptimal_ for all scenarios and replications.

### 2.8 Sensitivity test of E(*Y*) to deviations from normality

The presented formula for E(*Y*) and thus E(*G*) is exact under the assumption of normality. To test the sensitivity of our method to deviations from this assumption, we sampled random trait values from three different non-normal distributions: (1) A right-skewed t-distribution with *ν* = 10 and non-centrality parameter *λ* = 3, (2) a heavy-tailed t-distribution with *ν* = 10, and (3) a mix of two normal distributions with a shift in yield means of 3 dt/ha, resulting in a bimodal distribution.

All samples from all distributions were centered around the same mean. The scaling parameters for the t-distributions and the standard deviations for the mix of normals were chosen so that sample variances ranged between 1 and 20. For all scenarios, we investigated *n_i_* = 20, 60, and 100 and sampled sets *k* = 10 crosses. For the bimodal distribution, we divided the respective *n_i_* into two subsample sizes of 0.5 *n_i_* and drew sub-samples from the two normal distributions All scenarios were replicated 100 times.

For each cross in each set of *k* = 10 crosses, we calculated the sample mean and standard deviation, and used these parameters to predict E(*Y*) with Equation 6 under the assumption of normality. We selected the top *s_i_* = 5 individuals from the non-normal samples and calculated the mean over these 50 top individuals to get an estimate of the mean of the selected fraction. We then calculated the difference between this calculated mean of the total selected fraction and the predicted E(*Y*).

### 2.9 Evaluation of computation time for the optimization

To evaluate the computation time required for the optimization of the *n_i_*, we investigated a grid of the following scenarios: *k* = 20, 50, and 100 crosses, each tested with constant family sizes of *n_i_* = 20, 50, and 100. This resulted in total population sizes of *N* = 400, 1,000, 2,000, 2,500, 5,000, and 10,000. For each scenario, we used a constant *µ* = 92, randomly sampled $\sigma_{i}^{*2}$ ranging between 1 and 20, and a constant number of selected individuals *s_i_* = 5.

We recorded the elapsed computation time on a Linux server with 112 Intel^(R)^ Xeon Platinum 8276 CPUs. Each scenario was replicated 10 times and means of the elapsed computation times were calculated.

## 3 Results

**Figure 2:**
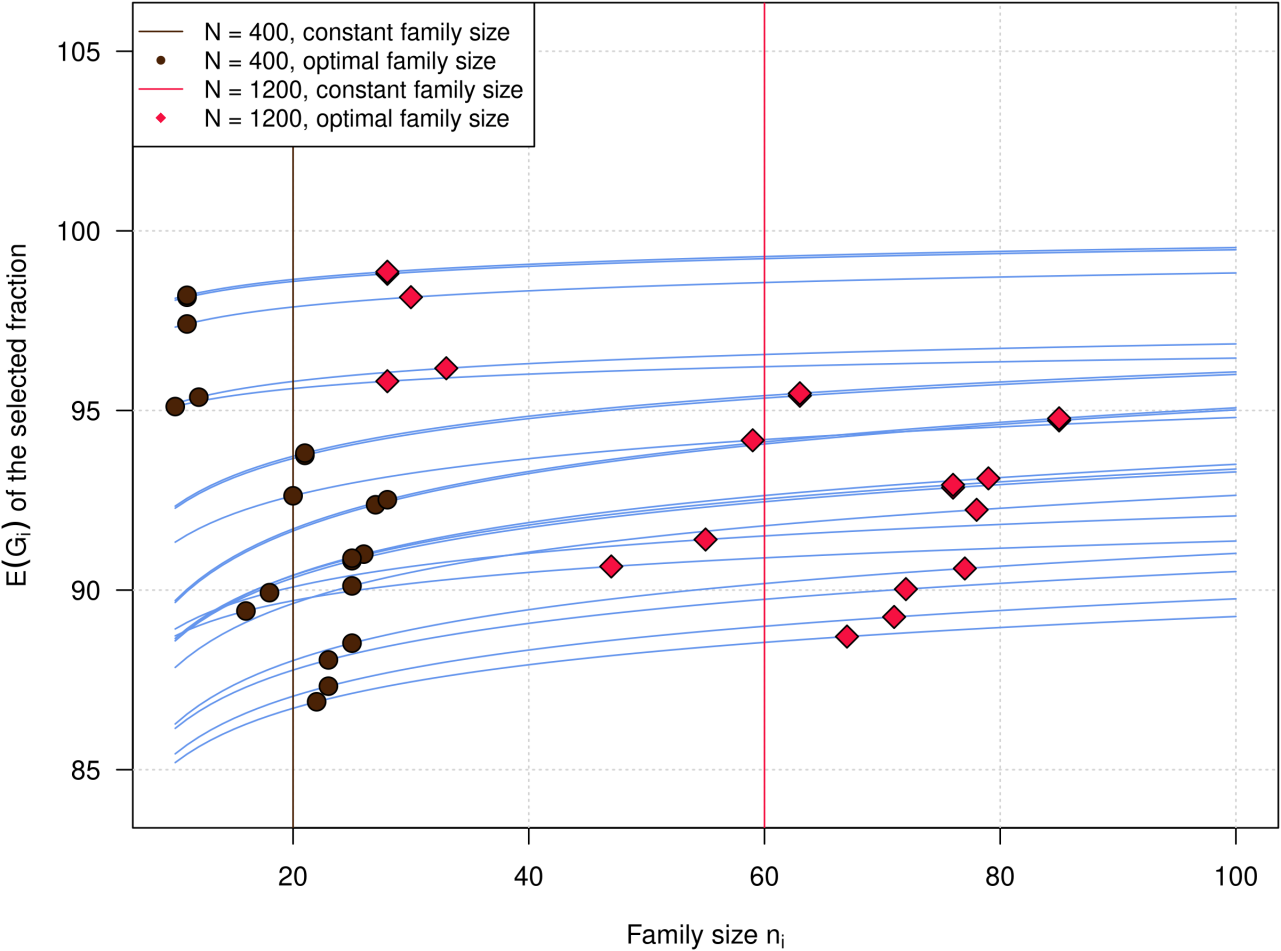
Expected family-specific genotypic values of the selected fraction in the barley crossing experiment as a function of *n_i_* for *s_i_*= 5. Vertical black and red lines represent constant family sizes *n_i_* for total population sizes of *N* = 400 and *N* = 1,200. Black dots and red diamonds represent optimal *n_i_* for total population sizes of *N* = 400 and *N* = 1,200.

**Figure 3:**
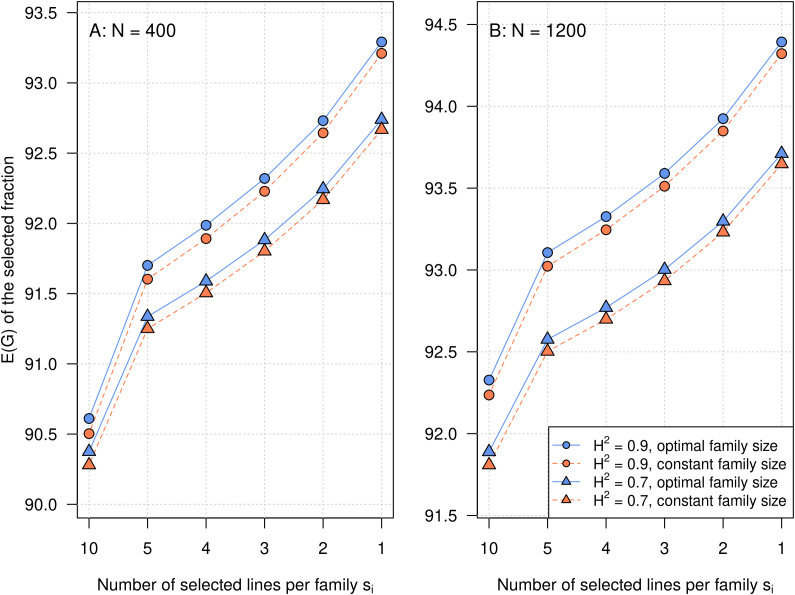
Expected genotypic value of the selected fraction in the barley crossing experiment. The total population size of *N* = 400 **(A)** or *N* = 1,200 **(B)** was allocated to 20 families such that the family size was either constant for all families (A: *n_i_* = 20, B: *n_i_* = 60) or optimal. The optimal family sizes were computed with automatic differentiation in Torch to maximize the selection gain. The heritabilities were *H*^2^ = 0.9 and *H*^2^ = 0.7.

The E(*G_i_*) functons of the crosses as well as the total E(*G*) target functions are always continuously increasing with increasing *n_i_*. We used the crosses of the experimental barley dataset to illustrate the properties of such E(*G_i_*) curves in **Figure 2**. The gradients of the E(*G_i_*) curves are always positive and constantly decreasing, approaching a value of 0. This is valid for arbitrary values of *µ_i_* and *σ_i_*^2^. As could be expected, the optimization algorithm assigned larger family sizes *n_i_* to crosses with a steeper gradient, i.e., a higher segregation variance. The variation of the optimal family sizes *n_i_* is increasing with increasing *N*. However, the gradients of the E(*G_i_*) curves already become comparatively shallow for *n_i_ >* 30–40 individuals. Beyond these values, only small increases in E(*G_i_*) and thus the total E(*G*) can be achieved by further increasing *n_i_*. With *N* = 1,200, most E(*G_i_*) curves are already approaching their asymptote. Because of the shallow gradients, the ranking of the crosses with respect to E(*G_i_*) is more or less the same over the whole investigated range of *n_i_*, and mostly determined by the cross means *µ_i_*. In the experimental dataset, the total E(*G*) is increasing with higher heritability, higher total population size *N*, and lower values of *s_i_* (**Figure 3**). Optimal family sizes result in higher E(*G*) than constant family sizes, but the differences were comparatively small, ranging between 0.06 and 0.11 dt/ha. The differences were larger for higher values of the heritability *H*^2^ and smaller values of the total population size *N*.

**Figure 4:**
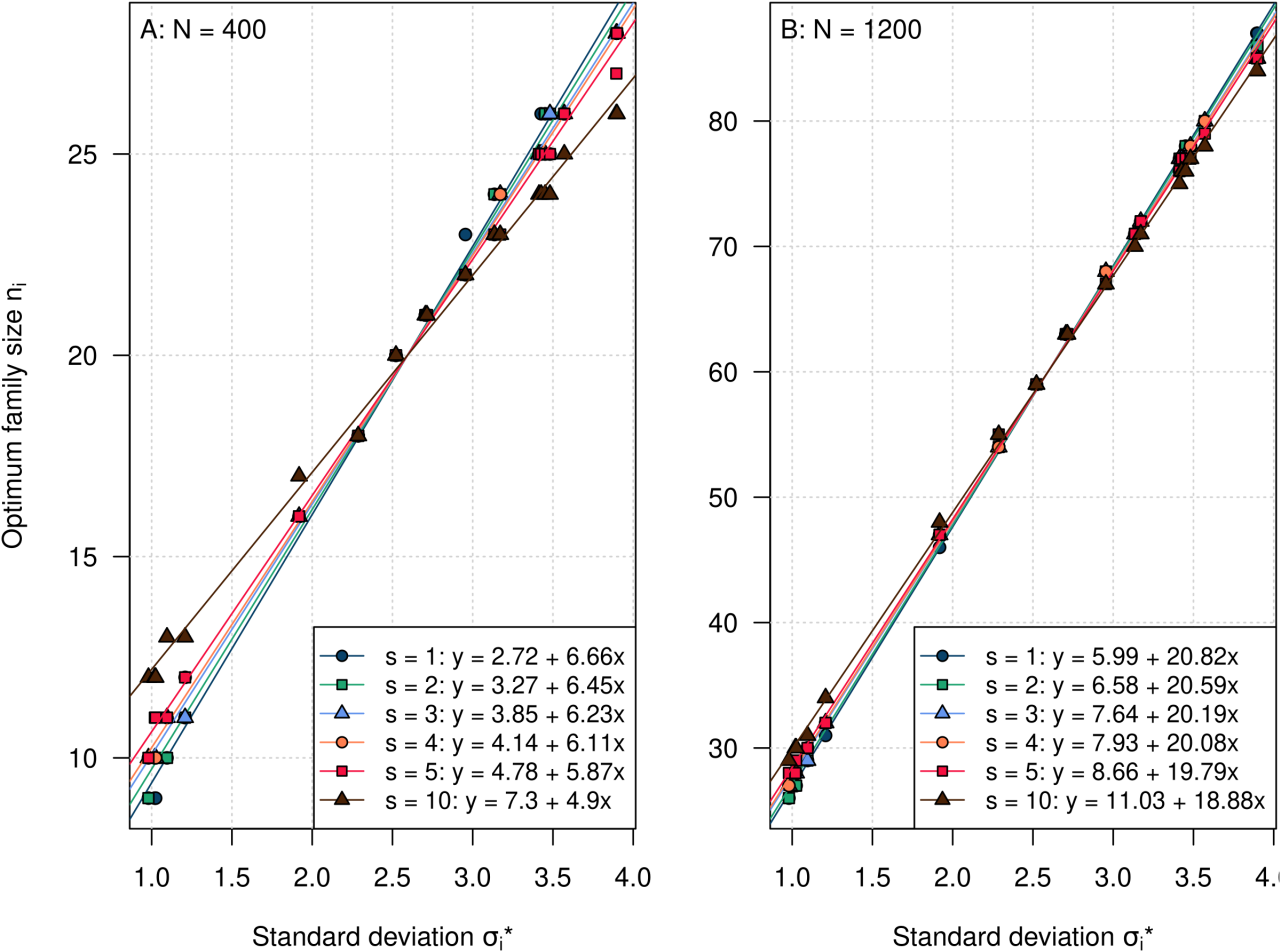
Linear regression of the optimal family sizes on the segregation standard deviation $\sigma_{i}^{*}$of a cross in the barley experiment. The total population size was *N* = 400 **(A)** or *N* = 1,200 **(B)**. Computations were carried out for a heritability of *H*^2^ = 0.9. The slope of the regression line is modified by the combination of *N* and *s_i_*.

We fitted regression models for investigating the relationship between the optimal family sizes *n_i_* and the segregation standard deviations (**Figure 4**). This relationship is linear, but dataset-specific, depending on the total population size *N* and the number of selected individuals *s_i_*, which determine the absolute values of the intercepts and slopes of the regression lines describing the association between the $\sigma_{i}^{*}$ and the *n_i_*. The slopes are steeper for *N* = 1,200 (**Figure 4B**) than for *N* = 400 (**Figure 4A**), and for smaller values of *s_i_*.* A* steeper slope corresponds to a higher variation in *n_i_*.* A* table with all optimal variable *n_i_* for the investigated scenarios of the experimental barley dataset is provided in the **Supplementary Materials** (**Supplementary Table 3**).

**Figure 5:**
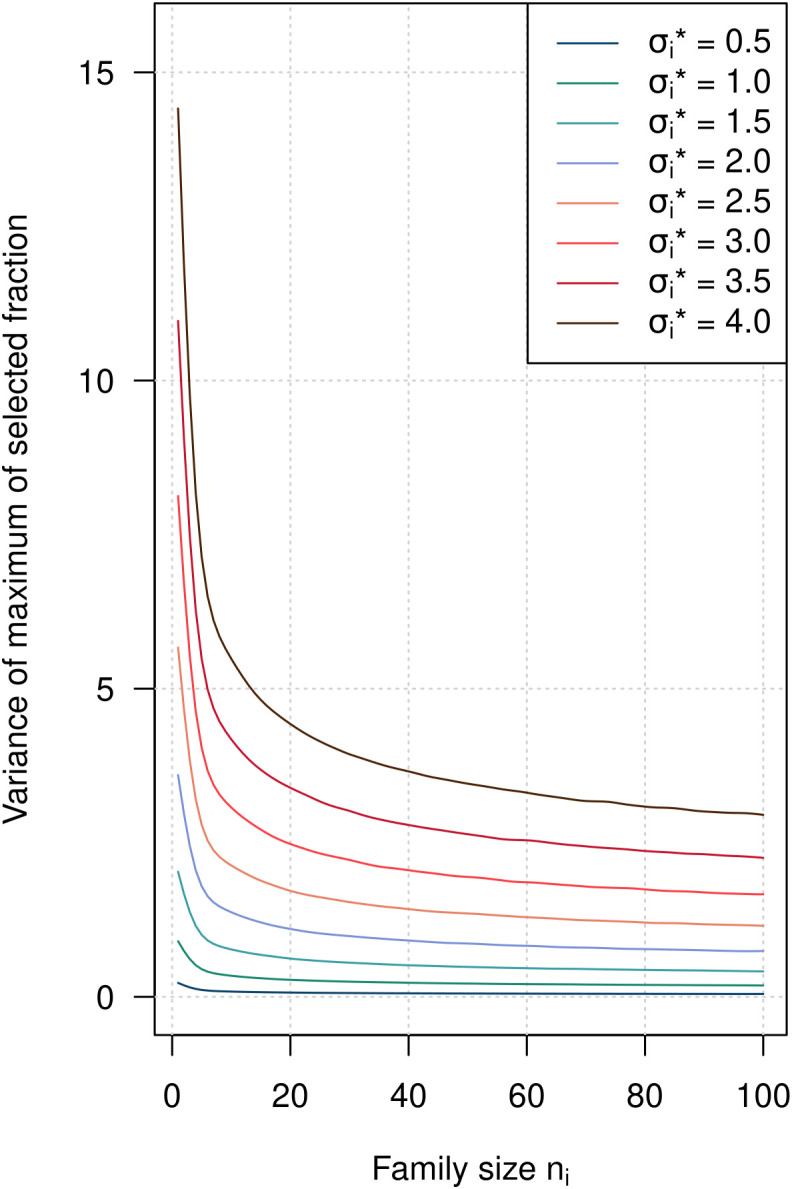
Variance of the maximum of the selected fraction as a function of *n_i_* for different values of the segregation standard deviation $\sigma_{i}^{*}$. The variance depends on $\sigma_{i}^{*}$ and decreases with decreasing *N*.

We investigated the effect of assigning more individuals *n_i_* to crosses with higher segregation standard deviations $\sigma_{i}^{*}$ on the reliability of the prediction of the selected fraction. In general, the variance of the predictions of the top order statistics of a cross is higher in crosses with higher segregation standard deviations $\sigma_{i}^{*}$, implying a lower reliability of the predictions. This is illustrated for the maximum order statistics of crosses with different values of $\sigma_{i}^{*}$ ranging between 0.5 and 4 (**Figure 5**). However, the variance of the top order statistic is rapidly decreasing with higher values of *n_i_*, indicating that allocating larger family sizes *n_i_* to crosses with high $\sigma_{i}^{*}$ increases not only the response to selection but also the reliability of the predictions.

**Figure 6:**
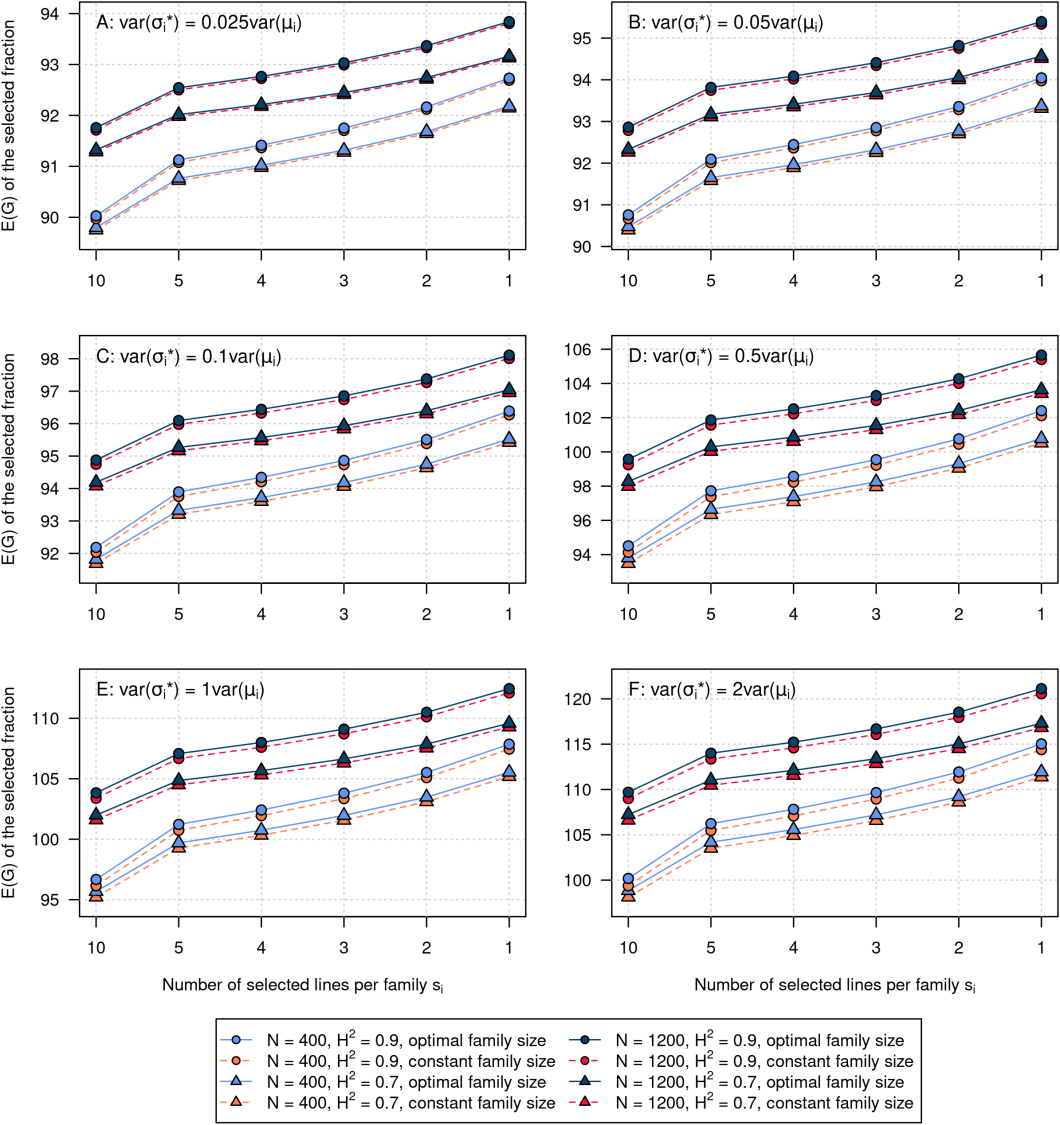
Expected genotypic value of the selected fraction in simulated datasets. Mean values over 10 replications. The total population size of *N* = 400 (light colors) or *N* = 1,200 (dark colors) was divided to 20 families such that the family size was either constant for all families (*n_i_* = 20 or *n_i_* = 60) or optimal. The optimal family sizes were computed with automatic differentiation in Torch to maximize the selection gain. The heritabilites were *H*^2^ = 0.9 and *H*^2^ = 0.7. Panels **(A–F)** show results for different values of the scaling factor *c*. **(A)**: *c* = 0.025, **(B)**: *c* = 0.05, **(C)**: *c* = 0.1, **(D)**: *c* = 0.5, **(E)**: *c* = 1, **(F)**: *c* = 2.

The observed trends for *N*, *H*^2^, and *s_i_* from the experimental dataset also held true for all 60 simulated datasets (**Figure 6**). For all investigated scenarios, the optimal family sizes outperformed the constant family sizes. The improvements with optimal progeny allocation ranged between 0.04 and 0.81 dt/ha. The improvements of E(*G*) are larger in datasets characterized by higher variability of the segregation variances, and smaller for larger values of *N*.

**Figure 7:**
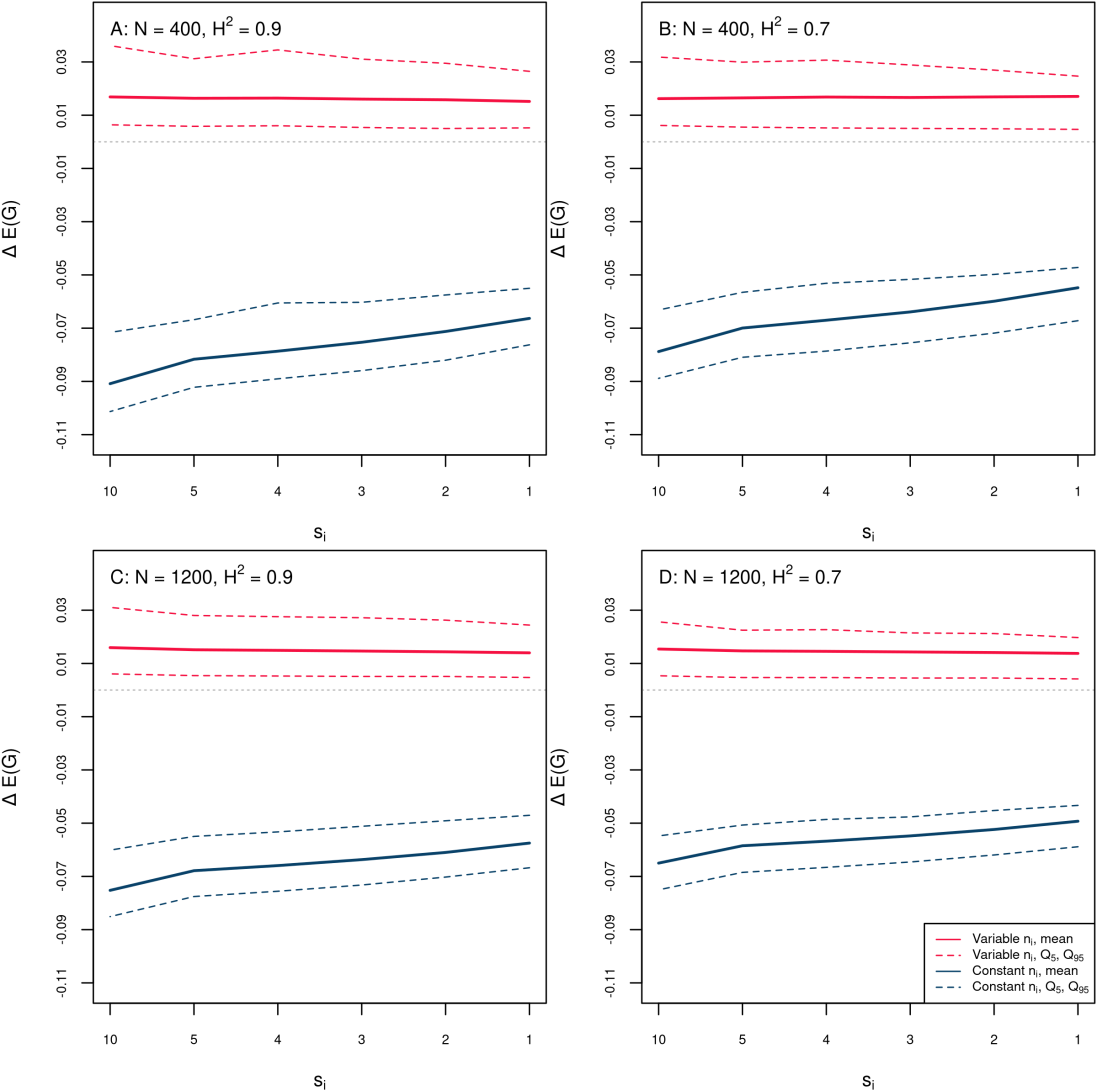
Results from the sensitivity test of the optimization to variance prediction error. Absolute differences Δ_var_ = E(*G*)_optimal_ − E(*G*)_suboptimal_ (red solid line: mean, red dotted lines: *Q*_5%_, *Q*_95%_) and Δ_const_ = E(*G*)_const_ − E(*G*)_suboptimal_ (blue solid line: mean, blue dotted lines: *Q*_5%_, *Q*_95%_) for simulated MAVE = 25%. A positive sign of Δ_var_ reflects a loss in E(*G*) compared to optimal *n_i_* derived from “true” segregation variances $\sigma_{i}^{*2}$ due to suboptimal allocation of *n_i_* derived from “noisy” simulated segregation variances. A negative sign of Δ_const_ reflects a gain in E(*G*) in compared to constant family sizes *n_i_*. Panels **(A–D)** show results for different values of the total population size *N* and heritability *H*^2^. **(A)**: *N* = 400, *H*^2^ = 0.9, **(B)**: *N* = 400, *H*^2^ = 0.7, **(C)**: *N* = 1200, *H*^2^ = 0.9, **(D)**: *N* = 1200, *H*^2^ = 0.7.

For all investigated levels of variance prediction error, the absolute differences Δ_var_ showed a positive sign and the Δ_const_ showed a negative sign (results for MAVE = 25% in **Figure 7**, results for MAVE = 10% and 5% in **Supplementary Figures 2** and **3**). A positive sign of Δ_var_ reflects a loss in E(*G*) compared to optimal *n_i_* derived from “true” segregation variances $\sigma_{i}^{*2}$ due to suboptimal allocation of *n_i_* derived from “noisy” simulated segregation variances and thus a reduction in the E(*G*) difference between variable and constant *n_i_*. These losses in the E(*G*) difference between variable and constant *n_i_* ranged on average between 0.43% and 0.75% for MAVE = 5%, between 2.01% and 2.33% for MAVE = 10%, and between 15.66% and 23.73% for MAVE = 25% (**Supplementary Table 2**). However, even at MAVE = 25%, the variable *n_i_* was always better than the constant *n_i_*, indicated by the negative sign of Δ_const_ (**Figure 7**).

**Figure 8:**
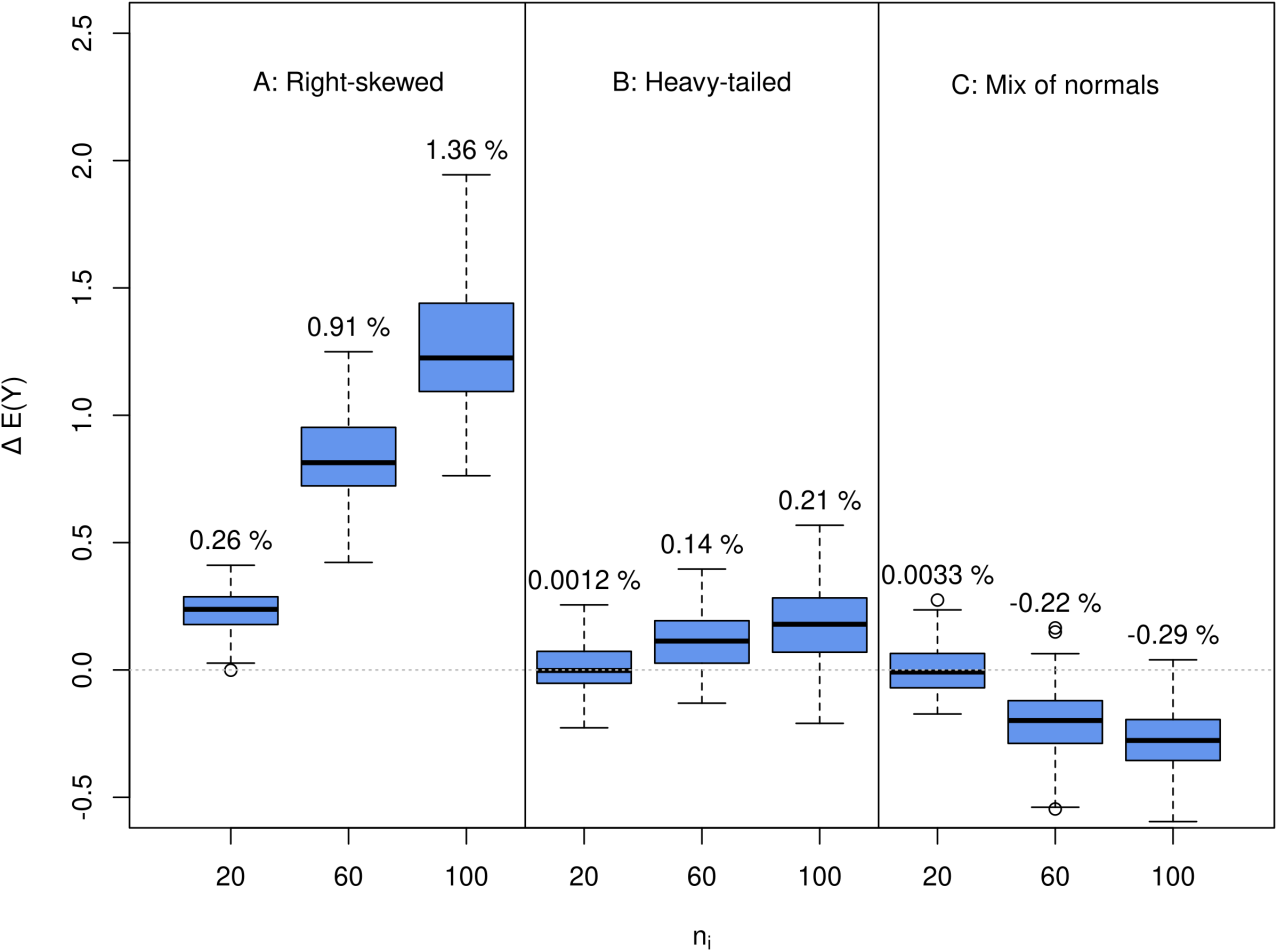
Boxplots over the 100 replications of the sensitivity experiment for deviations from normality with *k* = 10 crosses per replication. ΔE(*G*) are the differences between the observed means of the selected fractions of the simulated samples from the non-normal distributions and the predicted E(*Y*) from normal distributions with the same mean and variance as the non-normal distributions. Constant *n_i_* = 20, 60, and 100 and constant *s_i_* = 5 were investigated. Panels **(A–C)** show results for different non-normal distributions. **(A)**: Right-skewed t-distribution with *ν* = 10 and *λ* = 3, **(B)**: Heavy tailed t-distribution with *ν* = 10, **(C)**: Mix of two normal distributions with *µ*_1_ – *µ*_2_ = 3. Scaling parameters for the t-distributions and the standard deviations for the mix of normals were chosen so that sample variances ranged between 1 and 20.

The predictions of E(*Y*) and thus E(*G*) were robust against the investigated deviations from normality. The differences between the calculated means of the non-normal samples and the predicted E(*Y*) under the assumption of normality ranged between −0.29% and 1.36%, depending on the distribution (**Figure 8**). The greatest deviations were observed for the right-skewed t-distribution, followed by the bimodal distribution.

The computation time for running the optimization algorithm was linearly increasing in *N* (**Supplementary Figure 1**). The elapsed computation times ranged from 3.2 s for *N* = 400 to 86.9 s for *N* = 10,000. The effect of the number of crosses *k* on the computation time was negligible, as only the gradient of the cross that is allocated an additional individual is updated in every run.

## 4 Discussion

### 4.1 Comparison to related studies

In this study, we extend single-cross theory from quantitative genetics by applying theory of order statistics to derive a solution for evaluating the total response to selection, quantified as the expected genetic value E(*G*) in the following generation, for a breeding population consisting of a fixed set *k* crosses with varying cross means *µ_i_* and segregation variances $\sigma_{i}^{*2}$. The presented analytical solution is exact under the assumption of normality and correct specification of the cross parameters $\mu_{i}$ and $\sigma_{i}^{*2}$.

We used the derived function for E(*G*) as a target function for the optimization of progeny allocation to the *k* crosses under the constraints of a fixed total population size *N* and constant numbers of selected individuals *s_i_* per cross. We developed a greedy allocation algorithm based on the principle of automatic differentiation that yields the global optimum for the constrained integer allocation problem $\sum n_{i}$ = *N*, *n_i_*≥ *s_i_* (mathematical proofs for optimality are given in Equations 10–20 in Appendix 2).

There are a range of other studies available that also address the problem of optimal progeny allocation in breeding designs. Huehn (2005) presented solutions for optimal number of crosses and optimal finite family sizes *n_i_* with the aim of maximizing selection response. In contrast to our approach, the approach of Huehn can only handle fixed within-cross variances and does not provide exact solutions, but relies on the Burrows approximation.

Danguy des Déserts et al. (2023) investigated different criteria for between-cross selection in a one-generation simulation study with a special focus on different usefulness criteria. They maximized the sum of the products of the respective selection criterion multiplied by progeny sizes over crosses with linear programming or genetic algorithms and thus received an optimal progeny allocation for a fixed set of crosses. Similar to our approach, this study considers selection gain in the following generation and makes use of within-cross variance for progeny allocation. However, the study combines the problem of between-cross selection with the problem of progeny allocation, while our study focuses only on within-cross selection.

Oget-Ebrad et al. (2024) conducted an extensive simulation and validation study for evaluating the predictive ability of the usefulness criterion and investigated the impact of different sources of error on the prediction accuracy of segregation variances from genomic marker effects. They suggested that breeders should allocate more individuals to crosses with outstanding segregation variances to fully realize the variance potential of the crosses.

Moeinizade et al. (2019) optimized selection and mating in genomic selection with a look-ahead approach that considers only one progeny per parent pair. For allocating the number of progenies from a total budget to the crosses, they suggest a heuristic approach that assigns individuals proportional to the genetic diversity of the breeding parents. Both the study by Oget-Ebrad et al. (2024) and Moeinizade et al. (2019) suggest heuristics for progeny allocation, but do not provide exact optimal solutions.

In a follow-up study, Moeinizade et al. (2021) advanced their look-ahead optimization to a reinforcement learning-based algorithm that considers trade-offs between cost and time over multiple generations and can systematically learn resource allocation decisions under constraints.

Hamazaki and Iwata (2024) combined the optimization of parent selection, mate allocation, and progeny allocation over multiple generations by a complex algorithm that combines replicated future-oriented simulation with numerical black-box optimization with the aim of maximizing long-term selection gain.

In contrast to the approaches by Moeinizade et al. (2019); Moeinizade et al. (2021), and Hamazaki and Iwata (2024), our approach relies on closed-form expressions that allow for fast evaluation of the target function E(*G*) and a conceptually simple greedy gradient-based allocation algorithm that can be applied for short-term maximization of selection response without the need to run computationally expensive simulations over multiple breeding cycles.

### 4.2 Using order statistics for estimating the response to selection

The expectation of the selected fraction in Equation 5 is derived from the theory of order statistics. The order statistics approach is an alternative to using a truncated normal distribution. Using truncation of normal distributions, the selection differential of the selected fraction is (cf. Walsh and Lynch, 2018, p. 508)

(9) $$S_{i}=\varphi_{0,1}[{\text{Φ}}_{0,1}^{-1}(1-\alpha )]\frac{\sigma_{i}}{\alpha }$$

where *α_i_*= *s_i_/n_i_* is the selected fraction and *φ*_0,1_ and Φ_0,1_ are the density function and the quantile function of the standard normal distribution, respectively. The expectation of the selected fraction for a family is then


$$\text{E}(Y_{i})=\mu_{i}+H^{2}S_{i}.$$

Equation 9 does not correct for finite population size and consequently overestimates the selection differential for small population sizes. A well-known adjustment for this overestimation has been provided by Burrows (1972).

The solution presented in the present study requires the calculation of the expectation of order statistics from a normal distribution. At the time of the publication of the Burrows approximation, when computers were not widely available, it was necessary to resort to tabulated values for order statistics, which would have limited the practical use of the exact formula.

Nowadays, the components required for an order statistics approach are widely available in standard software and can be easily used. For example, in the R software (R Core Team, 2025) the function pnorm provides *φ* and integrate provides the numerical integration required for Equation 4. The calculations can be accomplished in a few lines of code with sufficient speed and numerical stability. Fast and efficient R code based on a Torch implementation of numerical integration for the calculation of order statistics is provided in the **Supplementary Materials**.

### 4.3 Independence of the total response to selection from the correlation of the cross means and the segregation variances

Under the assumption of fixed sets *µ_i_* and *σ_i_*^∗2^ and a fixed number of selected individuals *s_i_* per cross, the total response to selection over all crosses *R* does not depend on how the cross means *µ_i_* and the segregation variances $\sigma_{i}^{*2}$ are combined in the crosses. For cross *i*, the predicted response to selection is *R_i_* = *H*^2^*S_i_* = *H*^2^ [*E*(*Y_i_*) − *µ_i_*]. As a constant number $s_{i}$ of individuals is selected from each of the k families, the total response to selection R is the unweighted mean of the $R_{i}$: $R=1/k\sum_{k}R_{i}=H^{2}[1/k\sum_{k}E(Y_{i})-1/k\sum_{k}\mu_{i}]$. With $E(Y)=1/k\sum_{k}E(Y_{i})$ and $\mu =1/k\sum_{k}\mu_{i}$, we get $R=H^{2}[E(Y)-\mu ]$, which depends only on the grand mean μ but not on the means of the crosses $\mu_{i}$. For any set of *k* crosses with the same values of ${µ}_{i}\text{ and}\;\sigma_{i}^{*2}$, the total response to selection *R* is thus the same, irrespective of how ${µ}_{i}\text{ and}\;\sigma_{i}^{*2}$ are combined within the set of crosses. The optimal allocation of *n_i_* to the crosses with the objective to maximize *R* is thus independent of both the *µ_i_* and the correlation of the *µ_i_* and the $\sigma_{i}^{*2}$.

### 4.4 Optimization of *n_i_* by gradient ascent

The present study focuses on a scenario in which the response to selection is maximized as a function of the family sizes *n_i_* under the restriction of a fixed total number of individuals *N*.

The blue curves in **Figure 2** represent the family-specific expected genotypic values E(*G_i_*) for the *k* = 20 crosses of the experimental barley dataset. As the responses to selection *R_i_* and the E(*G_i_*) are independent of *µ_i_*, *µ_i_* only determines the location E(*G_i_*) curves on the *y*-axis, but not the gradients of the curves. The gradients and thus the marginal gains that can be attained by allocating more individuals to a family only depend on *s_i_*, which are fixed by the breeder, and on the $\sigma_{i}^{*}$ of the crosses.

The optimal allocation of *n_i_* to the families will therefore in some way be proportional to the $\sigma_{i}^{*}$. The exact proportions are determined by the absolute values of the gradients of the E(*G_i_*) curves that depend on *N* and *s_i_*.

The gradients of the E(*G_i_*) curves are continuously increasing with increasing *n_i_* and asymptotically approaching a plateau. The plateau represents the maximum possible E(*G_i_*) with infinite family size. The gradients of the curves are always positive and constantly decreasing in absolute value with increasing *n_i_*. Consequently, it is impossible to miss out on later catch-up effects when always assigning the next individual to the cross with the current largest gradient.

Mathematically speaking, the functions for E(*G_i_*) curves are monotonously increasing in *n_i_* and concave. Under these conditions, a greedy allocation algorithm that always assigns the next individual to the cross with the current largest gradient yields the global optimum for the constrained integer allocation problem $\sum n_{i}=N$, $n_{i}\geq s_{i}$. Mathematical proofs for monotonicity and concavity of E(*G_i_*) that should hold for i.i.d. genotypic values sampled from continuous trait distributions with finite means are provided in Equations 10–16 (monotonicity) and Equations 17–20 (concavity) in Appendix 2.

Building a numerical optimization algorithm on the concept of gradient ascent was therefore the logical rationale. The free open-source library Torch with its functionality for automatic differentiation and R interface offered a fast and easy-to-use implementation for iterative allocation of individuals to the crosses (Paszke et al., 2017, 2019; Falbel and Luraschi, 2025).

We implemented all required functions for the prediction of E(*G_i_*) from Equations 1 to 8 in Torch and used its functionality for automatic differentiation for calculating the gradients that we used as a criterion for the allocation of the *n_i_*. We share the R code including a stand-alone example for the experimental barley dataset in the **Supplementary Materials**. The R code also generates figures that show both the stepwise allocation of individuals to crosses and the gradients of E(*G_i_*) over iterations for all investigated crosses. The workflow of the optimization is described in the commented pseudo-code in **Appendix 1**. Users can plug in estimates of cross means *µ_i_* and segregation variances $\sigma_{i}^{*2}$ into the provided R code and specify *N* and *s_i_* to obtain a vector of optimal values for *n_i_* as output.

### 4.5 Effect of optimized *n_i_* in the experimental dataset

We applied the newly developed optimization algorithm on the experimental barley dataset for two different values of *N*, six different values of the number of selected individuals *s_i_*, and two levels of *H*^2^ (**Figure 3**). As expected, E(*G*) increased with higher heritability, higher total population size *N*, and lower values of *s_i_*, corresponding to higher selection intensity.

Under all scenarios, the optimal variable family sizes derived with the new algorithm outperformed the constant family sizes, although absolute values of the differences were comparatively small. An explanation for this can be found in **Figure 2**. Even though the experimental dataset consisting of both donor × elite crosses and elite × elite crosses can be expected to be quite extreme with respect to the differences in segregation variances between the crosses, the gradients of the E(*G_i_*) curves are comparatively shallow. As a consequence, the ranking of the E(*G_i_*) curves is stable with increasing *n_i_*. Even larger increases in *n_i_* only result in comparatively small gains in E(*G_i_*). Moreover, under the restriction of a fixed total population size *N*, gains in E(*G_i_*) that are attained by increasing *n_i_* in families with steeper gradients, i.e., higher segregation variances, compared to constant family sizes are always bought at the expense of losses in E(*G_i_*) in families with shallow gradients, i.e., lower segregation variances, which are incurred by decreasing *n_i_* compared to constant family sizes.

Disruptive increases by optimizing *n_i_* can therefore not be expected with fixed total population size *N* and without between-cross selection. However, one important finding is that as long as the gradients at the point of constant *n_i_* differ, the gains in E(*G_i_*) with the optimal family sizes will always overcompensate the losses. In the unlikely worst case, when all gradients are the same, the gradient ascent algorithm is expected to end up with constant *n_i_*.

We tested the computation times required for the optimization of *n_i_* for *N* = 400 to up to *N* = 10,000. The computation time is linear in *N* and independent of the number of crosses *k*. For the experimental dataset, the optimization took approximately 3 s. For *N* = 10,000, it took 86 s.

Given that the algorithm presented here is fast and easy to implement and a reliable tool for practical applications, we conclude that it is worthwhile to apply in almost all breeding scenarios.

It can be expected that small improvements in E(*G*) will accumulate over cycles to significant improvements in the long term, especially in breeding scenarios that combine optimized progeny allocation with between-cross selection and management of long-term diversity. This is confirmed by a simulation study that reported that optimized allocation of progeny resulted in almost 10% higher genetic gain within four generations compared to constant allocation (Hamazaki and Iwata, 2024).

### 4.6 Relationship between segregation variances and optimal *n_i_*

The optimal family sizes *n_i_* determined with the gradient ascent algorithm are approximately proportional to the segregation standard deviations (**Figure 4**). A linear dependency seems to approximate the relationship well for all investigated parameter combinations.

The regression lines can be used for determining the approximate number of allocated individuals *n_i_* by plugging in values of $\sigma_{i}^{*}$. For example, for the scenario *N* = 400, *s_i_* = 5, a cross with a segregation standard deviation of $\sigma_{i}^{*}=1.5$ would get ≈ 14 individuals assigned, while a cross with $\sigma_{i}^{*}=3$ would get ≈ 22 individuals. A table with all optimal variable *n_i_* for the investigated scenarios of the experimental barley dataset can also be found in the **Supplementary Materials** (**Supplementary Table 3**).

The intercepts and slopes of the regression lines describing the association between the $\sigma_{i}^{*}$ and the *n_i_* change with different values of *N* and *s_i_*.

The increase of the intercepts with increasing *N* reflects that the average *n_i_* per cross is increasing. The increase of the slopes with increasing *N* suggests that the ratios of the *n_i_* between crosses with low and high $\sigma_{i}^{*}$ are increasing.

The same trends are observed when *s_i_* decreases at a fixed level of *N*, i.e., when the selection intensity is increasing. We conclude that the intercept generally reflects the evenness of the distribution of the *n_i_*, whereas the slope of the regression line reflects the ratio of the *n_i_* between the crosses.

At *N* = 400, the differences between the slopes of the regression lines increase faster with decreasing *s_i_* than at *N* = 1,200. This suggests that for large *N*, the effect of decreasing *s_i_* on the distribution of the *n_i_* weakens and results in smaller differences in the distribution of the *n_i_* between different values of *s_i_*. This is probably related to the fact that for the same values of *s_i_*, the differences between the selected fractions *α_i_* are getting smaller with higher *N*.

Another explanation might be that the gradients of all E(*G_i_*) curves are more shallow at *N* = 1,200 than at *N* = 400 (**Figure 2**). It can therefore be expected that the gradient differences that are caused by different values of *s_i_* are becoming smaller with large *N*, resulting in smaller differences in the ratios of the *n_i_*.

We conclude that there is no single heuristic criterion for the optimization of *n_i_* that is applicable to every dataset. Under different parameter settings, particularly different ratios of the $\sigma_{i}^{*}$, optimal allocation of *n_i_* requires scenario-specific optimization.

### 4.7 Increased reliability of predictions with optimal *n_i_*

Apart from achieving a higher value of E(*G*) with optimal family sizes, allocating more individuals to crosses with higher segregation variances has another advantage. The variance of the predicted values E(*G_i_*) for individual crosses is increased with higher segregation variance. We illustrate this in **Figure 5** with the variance of the highest order statistic of crosses with segregation standard deviations $\sigma_{i}^{*}$ ranging between 0.5 and 4. A higher $\sigma_{i}^{*}$ results in a higher variance of the top order statistic. For high values of $\sigma_{i}^{*}$, the variances are rapidly decreasing with increasing *n_i_* and continue to decrease up to *n_i_*= 100. For low values $\sigma_{i}^{*}$, the decrease in the variance of the top order statistic is much smaller and the variances quickly reach a plateau.

Breeders are probably familiar with this concept from dealing with the standard error of cross means SEM that is also a function of the within-cross variance and *n_i_* ( $\text{SEM}=\sqrt{\sigma_{w}^{2}/n_{i}}$) where the same principles apply.

Optimization of progeny allocation will increase *n_i_* in crosses with higher $\sigma_{i}^{*}$ and decrease *n_i_* in crosses with lower $\sigma_{i}^{*}$. As the variance of the selected top order statistics is decreasing faster with increasing *n_i_* in crosses with higher $\sigma_{i}^{*}$, and is increasing with decreasing *n_i_* in crosses with lower $\sigma_{i}^{*}$, we can expect that the overall variance of both E(*G_i_*) and E(*G*) will be reduced by the optimization, resulting in more reliable predictions.

### 4.8 Transferability of observed trends to other datasets

Irrespective of the investigated set of crosses, the location of the E(*G_i_*) curves is determined by the *µ_i_*, and the gradient by the $\sigma_{i}^{*}$. The gradients are always positive and decreasing, approaching 0 at the plateau. Optimizing *n_i_* by gradient ascent will always pay off as long as the gradients of the E(*G_i_*) curves differ at the point of constant *n_i_*. To investigate the transferability of the observed trends to datasets with different ratios and variances of the $\sigma_{i}^{*}$, we created 60 simulated datasets. The *µ_i_* and $\sigma_{i}^{*}$ were sampled from normal distributions with 10 replications per scenario (**Figure 6**). The variance of the *µ_i_* was the same as in the experimental dataset. The variance of the $\sigma_{i}^{*}$ was derived from the variance of the *µ_i_* by multiplication with a factor ranging between 0.025 and 2. For comparison, in the experimental dataset the variance of the $\sigma_{i}^{*}$ was approximately 0.05 × the variance of the *µ_i_*. Values in a range between 0.02 and 0.11 have also been observed in a study on wheat (Danguy des Déserts et al., 2023).

We expect that in real-life breeding programs with diverse donor × elite crosses, the correlation between *µ_i_* and $\sigma_{i}^{*}$ will be negative in most cases. However, as has been shown above, the response to selection is completely independent from the correlation of *µ_i_* and $\sigma_{i}^{*}$ in a fixed set of crosses. The assortment of *µ_i_* and $\sigma_{i}^{*}$ in the simulated datasets was therefore completely random. The trends for *H*^2^, *N*, and *s_i_* were consistent with the experimental dataset. For all scenarios, optimal family sizes outperformed constant family sizes. We therefore expect that the conclusions on the optimization of the family sizes *n_i_* are transferable to a wide range of arbitrary breeding populations. Optimizing *n_i_* by gradient ascent should be beneficial in all datasets characterized by different cross means and segregation variances.

Optimal progeny allocation yielded the largest improvements with a small total population size *N*, a high ratio $\text{Var}(\sigma_{i}^{*})/\text{Var}(\mu_{i})$, and low selection intensities corresponding to large values of *s_i_*. All these factors seem to add up and interact. A greater effect of optimal progeny allocation with higher ratios of $\text{Var}(\sigma_{i}^{*})/\text{Var}(\mu_{i})$ was also observed by Danguy des Déserts et al. (2023).

As the optimization always yielded higher absolute improvements for *N* = 400 than for *N* = 1,200, we conclude that small-scale breeding programs will profit more from optimization of *n_i_* than large-scale breeding programs. An explanation for this effect might be that the gradients of the E(*G_i_*) curves are steeper and the differences between gradients are larger for smaller *N* (**Figure 2**). The highest absolute improvements were observed in breeding populations with high ratio $\text{Var}(\sigma_{i}^{*})/\text{Var}(\mu_{i})$ and low selection intensities. In these settings, the improvements in E(*G*) were almost eight times higher than for the experimental dataset. We therefore consider pre-breeding programs or early phases of breeding pool establishment as the most promising areas of application.

### 4.9 Sensitivity to variance prediction error

The optimization presented here requires estimates of the segregation variances of the crosses. The type of the investigated population, e.g., SSD lines or doubled haploid lines, and the number of generations of intermating before selfing determine the prediction of the segregation variances of the crosses. Different approaches for obtaining such estimates from marker effect estimates from genomic selection have been published for crosses consisting of inbred lines generated by several generations of selfing and doubled haploid lines (Zhong and Jannink, 2007; Bonk et al., 2016; Osthushenrich et al., 2017; Lehermeier et al., 2017). The derivations of the segregation variances are based on similar assumptions, and in most cases, they provide similar variance estimates.

The prediction accuracy for segregation variances with current methods is currently low to moderate (Yao et al., 2018; Adeyemo and Bernardo, 2019; Neyhart and Smith, 2019; Wolfe et al., 2021; Rembe et al., 2022; Oget-Ebrad et al., 2024), indicating that there is a high potential for improvement by further research. Potential sources of inaccuracy for prediction of segregation variances are, among others, low quality of phenotypic data or small training set sizes for estimating marker effects, choice of inappropriate statistical models for estimation of marker effects, and recombination frequencies from linkage maps that do not exactly match the recombination patterns of the investigated breeding population. These sources of prediction error will affect not only the prediction of the segregation variances but also any method of genomic prediction, simulation, or cross planning that relies on the same data resources. We therefore emphasize the importance of using of high-quality data.

It can be expected that future research will provide more accurate estimates of the segregation variance. The approach presented here will work with arbitrary estimates of the segregation variances. Improved variance estimates can replace the current state-of-the-art estimates that were employed here.

Nevertheless, we tested the robustness of our optimization approach against three different levels of MAVE that amounted to MAVE = 5%, 10%, and 25% of the absolute cross-specific segregations variances $\sigma_{i}^{*2}$. For all investigated levels of the variance prediction error, we observed a loss in the E(*G*) difference between variable and constant *n_i_* due to the suboptimal allocation of *n_i_* (results for MAVE = 25% in **Figure 7**, and results for MAVE = 10% and 5% in **Supplementary Figures 2** and **3**). As expected, the loss was lowest for MAVE = 5%, the magnitude being approximately 0.5% of the original difference, followed by approximately 2.2% for MAVE = 10% and 16%–24% for MAVE = 25% (**Supplementary Table 2**).

This indicates that higher levels of variance prediction error can considerably diminish the gains from optimization of *n_i_*. However, the E(*G*) achieved with suboptimal variable *n_i_* was still always better than E(*G*) with constant *n_i_* (**Figure 7**). We therefore conclude that optimization is also worthwhile under variance prediction error.

### 4.10 Robustness to deviations from normality

The analytical solution for the prediction of order statistics and thus E(*G*) is exact under the assumption of normality. This is a frequent theoretical assumption in quantitative genetics that is never perfectly met in reality. Depending on the investigated trait and its genetic architecture, heavy-tailed or skewed trait distributions can occur, e.g., in the presence of a single major gene or a few QTL with large effects.

We tentatively simulated three different non-normal trait distributions to check the sensitivity of our approach to deviations from the normal distribution: a right-skewed distribution, a heavy-tailed distribution producing more outliers in the extremes, and a bimodal distribution constructed from a mix of two normal distributions.

The predictions of E(*Y*) that are the basis of E(*G*) were pretty robust against the investigated deviations from normality. The differences between the calculated means of the non-normal samples and the predicted E(*Y*) under the assumption of normality ranged between −0.29% and 1.36%, depending on the distribution (**Figure 8**). The greatest absolute deviations were observed for the right-skewed distribution, followed by the bimodal distribution.

Deviations from the normal distribution will affect E(*Y*) if the respective distribution produces more or fewer extreme values than a normal distribution with the same mean and standard deviation on the tail where the truncation threshold for selection lies.

In **Figure 8**, we can see that prediction with our formulas underestimates E(*Y*) for the right-skewed distribution (A) and the heavy-tailed distribution (B). For these distributions, the algorithm would assign too few individuals to crosses with high segregation variance, especially for higher levels of *N*. The opposite is true for the bimodal distribution (C). Here, we tend to overestimate the observed E(*Y*) with our formulas. In this case, we can expect that the optimization algorithm would assign too many individuals to crosses with higher segregation variance.

In general, we can expect that the probability of observing extreme values in a sample increases with increasing *n_i_*. All observed trends were consequently increasing with larger *n_i_*.

We conclude that breeders should try to collect data on the assumed trait distributions. If a more realistic continuous density function for the real trait distribution is known, it can be plugged into Equation 4 instead of the normal distribution.

### 4.11 Applications in breeding programs

The results of the present study suggest that optimizing family sizes *n_i_* is advantageous when the segregation variances differ between crosses. **Figure 6** shows that optimizing *n_i_* leads to higher absolute gains in scenarios with large differences in segregation variances. In breeding programs that mainly rely on elite × elite crosses, the gains that can be achieved by optimizing *n_i_* will therefore probably be smaller than in breeding programs introgressing novel diversity from donor genotypes. We therefore see pre-breeding programs with a high ratio of $\text{Var}(\sigma_{i}^{*})/\text{Var}(\mu_{i})$ as a promising field of application for this optimization method.

The optimization algorithm maximizes selection gain in the short term. This is of relevance for generating candidates for variety testing from the cycles of long-term recurrent breeding programs. For this application, the presented optimization algorithm is a computationally efficient tool to make the most of every cross. Optimal progeny allocation that maximizes selection gain in general increases the efficiency of selection. As a consequence, it will have negative effects on genetic diversity when used as a stand-alone method, e.g., in best × best crosses or with criteria as the usefulness. In multi-cycle recurrent selection schemes, we can expect that repeated selection with optimized *n_i_* will increase selection gain faster in comparison to suboptimal *n_i_* allocation, but because of the increased efficiency of selection, we will also run out of genetic diversity faster, which will level out initial gains in the long term. We therefore suggest to combine the optimization of *n_i_* in within-cross selection with methods of parent selection and mate allocation to balance efficient selection with maintenance of long-term diversity.

For this purpose, the constraint of a constant number of selected individuals *s_i_* per cross, employed in the present study for keeping the illustration of the newly developed methods as simple as possible, needs to be relaxed. From the programming perspective, this is trivial. Instead of passing a single integer value of *s_i_*, breeders can pass a vector of variable *s_i_* to the optimization algorithm. Variable *s_i_* could either be optimized for maximizing short-term selection gain, e.g., with the method of Melchinger et al. (2024), or with respect to finding an optimal balance of selection gain and long-term genetic diversity. For this purpose, methods like optimum contribution selection can be applied that determine the optimal contribution of each parent to the following generation. From the derived fractions for each parent, a vector of variable *s_i_* can be derived for optimization.

For the introduction of the new methodology, we focused only on within-cross selection in this paper. As can be seen from **Figure 2**, **3** and **6**, the gradients of the E(*G_i_*) curves as a function of *n_i_* are pretty shallow, resulting in comparatively small gains in E(*G*) in our investigated scenarios. This is an important finding that should be reported, highlighting again the importance of selecting parents that result not only in crosses with high expected mean performance but also in complementary crosses with high segregation variances.

In general, the gains incurred by optimal progeny allocation were largest in scenarios with low *N* (**Figure 3** and **6**). Looking at **Figure 2**, we can see that with *N* = 400, the gradients of the E(*G_i_*) curves are much steeper than the gradients with *N* = 1,200. At the level of *N* = 1,200, some of the curves are already approaching the plateau. This has several implications. First, it means that small-scale breeding programs with limited resources will profit most from optimizing *n_i_*. Second, it means that increasing *N* beyond a level where the marginal gains fall beyond a certain threshold is inefficient.

Where this threshold lies is specific for the respective breeding program and can be determined by experienced breeders. In general, considering the simplicity and low computational costs of running the optimization, we think it is worthwhile to run it in all scenarios, especially in commercial settings where making the most of limited resources is mandatory, and the algorithm always gives the global optimum, as has been shown in **Appendix 2**.

However, we explicitly recommend to determine in every cycle of a breeding program the program-specific maximum sensible population size *n_i_*. The closed formulas for prediction of E(*G_i_*) allow for the quick evaluation of a wide range of *n_i_* values. By setting a program-specific threshold for the minimum marginal gain that needs to be realized by adding an additional individual to the respective cross, breeders can determine maximum sensible family sizes *n_i_* for all planned crosses beyond which adding more individuals is inefficient. This can be considered the optimal progeny allocation without resource constraints. Whenever the feasible *N* determined by resource restriction lies below the sum of these maximum sensible *n_i_*, optimization is definitely worthwhile.

In every cycle of a recurrent selection scheme, both the variance of $\sigma_{i}^{*}$ and the variance of *µ_i_* will decrease, implying that the maximum sensible *N* will decrease. Excess testing capacities in advanced cycles can then be re-allocated to other branches of the breeding program or can be used for evaluating more crosses.

From these considerations, we conclude that an efficient application of optimal progeny allocation is highly breeding program-specific and breeding goal-specific. It needs to be investigated in the context of many other parameters, such as the overall dimensions of the breeding program, the diversity of parents, and the strategy of mate allocation. This is beyond the scope of the present study.

Optimal progeny allocation in the context of multi-cycle schemes that balance the trade-off between selection gain and long-term diversity has also been attempted in previous studies (Moeinizade et al., 2019, 2021; Hamazaki and Iwata, 2024). These studies are usually look-ahead strategies based on computationally expensive simulations that design breeding programs over multiple generations and maximize future selection gain in advanced cycles under diversity and budget constraints. The reasoning behind these approaches is very sound. However, according to our experiences in real-life breeding, it is frequently the case that a fraction of the initially planned crosses fails or does not produce enough viable offspring for testing or further crosses. In these situations, the new methods introduced in this study might provide a flexible and fast alternative for re-adjusting the cross plan.

### 4.12 Further research needs

The focus of the present study was on the extension of single-cross theory from quantitative genetics to multiple crosses, and on the development of an optimization algorithm for optimal progeny allocation under constraints.

To explore the potential of the newly developed methods in large scale recurrent breeding programs, extensive multi-cycle simulation studies based on real-life datasets with larger dimensions will be useful. This will also allow for further robustness testing.

We expect that the small improvements in E(*G*) observed in all investigated datasets will accumulate over time when optimal progeny allocation is combined with methods for between-cross selection, management of genetic diversity, and appropriate resource allocation over cycles. Such simulation studies should be run over a grid of a wide range of parameters and crossing schemes and tested against other strategies. This work is in progress, but beyond the scope of the present study.

Another field that merits further research is the impact of using variable *s_i_* in combination with optimal progeny allocation. Approaches for the management of genetic diversity and inbreeding such as optimum contribution selection will make it necessary to relax the constraint of constant *s_i_* per cross. This can be easily achieved by passing a vector of variable *s_i_* to the optimization algorithm. However, it might also be necessary to consider additional constraints such as minimum *n_i_* and *s_i_* per cross. As a starting point, the family sizes could be initialized with max(min(*n_i_*), min(*s_i_*)), and bounds for both *n_i_* and *s_i_* could be included that need to be checked in every iteration of the algorithm.

As shown by Melchinger et al. (2024), selection gain can also be improved by optimizing the selected fractions of individual crosses. The optimization of *s_i_* could in theory also be achieved by automatic differentiation. However, this is not as straightforward as optimal progeny allocation, as E(*G*) is not monotonous in *s_i_*. It is also not independent from the cross means. It can be expected that the optimal *s_i_* is in some way proportional to $\mu_{i}+\sigma_{i}^{*}$. However, as can be deduced from the regression lines in **Figure 4**, optimal *n_i_* and *s_i_* are mutually dependent, which will make simultaneous optimization necessary to reach an optimum that is near the global optimum.

It can be expected that there will be a greater trade-off between maintenance of genetic diversity and maximization of E(*G*) as a function of *s_i_* than with optimal assignment of *n_i_* only, especially in sets of crosses with a low ratio of $\text{Var}(\sigma_{i}^{*})/\text{Var}(\mu_{i})$. In these sets of crosses, the crosses with high means will probably get assigned a greater contribution to the following cycle. We plan extensions of our algorithm and further investigations in this direction.

### 4.13 Conclusions

The derived formulas for predicting the expectation, the response to selection, and the genetic value of the total selected fraction from multiple crosses allow for quick and efficient evaluation of fixed sets of crosses. The derived optimization algorithm for progeny allocation yields the global maximum of the genetic value of the selected fraction under the assumption of fixed sets of crosses and finite population size *N*. The optimal allocation of family sizes is independent of the cross means and shows a linear relationship with the segregation standard deviations. This relationship is dataset-specific and modified by the combination of the total population size and the sizes of the cross-specific selected fractions. It thus cannot be derived by simple heuristics. Breeders can directly use the provided R implementation of the developed methods to maximize short-term selection gain in their breeding programs without need of computationally expensive simulations.

## Data Availability

The experimental dataset has been previously published. The raw genotypic data is confidential material from commercial breeding partners. The relevant parameters for reproducing the outcomes of the present study are included in the **Supplementary Material**. Requests to access these datasets should be directed to eva.herzog@agrar.uni-giessen.de.

## Appendix 1: Pseudocode for the greedy optimization algorithm

Algorithm 1. Greedy allocation of the total sample size *N* to k crosses

## Appendix 2: Mathematical proofs

If the family-specific E(*G_i_*) is increasing and concave in *n_i_*, the presented optimization algorithm that always assigns the next individual to the cross with the current largest gradient yields the global optimum for the constrained integer allocation problem $\sum n_{i}=N\text{, }n_{i}\;\geq \;s_{i}$.

To prove this, it is sufficient to show that

(10)
$$E(Y_{i}{)}_{n_{i}}\;\geq \;E(Y_{i}{)}_{n_{i}+1}$$

and that

(11)
$$E(Y_{i}{)}_{n_{i}+1}-E(Y_{i}{)}_{\text{ni}}\leq E(Y_{i}{)}_{\text{ni}}-E(Y_{i}{)}_{n_{i}-1}$$

Let the genotypic values of the individuals of a cross *i* be i.i.d. random variables *X_i1_*, *X_i2_*… with a finite mean *μ*. We denote the order statistics for population size *n_i _* ≥ *s_i _* by ${X_{\left(1\right)}}^{\left(n_{i}\right)}\text{ ≤ … ≤ }X_{\left(n_{i}\right)}^{\left(n_{i}\right)}$. The order statistics for population size *n_i _*+ 1 ≥ *s_i _* are denoted by $X_{\left(1\right)}^{\left(n_{i}+1\right)}\leq \text{ … ≥ }X_{\left(n_{i}+1\right)}^{\left(n_{i}+1\right)}$.

Using Equation 5, the expectation of the selected fraction consisting of a fixed number *s_i_* of the top selected individuals from a cross *i* with family size *n_i _* ≥ *s_i _* is then

(12)
$$E(Y_{i}{)}_{n_{i}}=\frac{1}{s_{i}}\sum_{r=n_{i}-s_{i}+1}^{n_{i}}E({X_{i(r)}}^{\left(n_{i}\right)})$$

The expectation of the selected fraction consisting of a fixed number *s_i_* of the top selected individuals from a cross *i* with family size *n_i _* + ≥ *s_i _* is

(13)
$$E(Y_{i}{)}_{n_{i}+1}=\frac{1}{s_{i}}\sum_{r=n_{i}+1-s_{i}+1}^{n_{i}+1}E({X_{i(r)}}^{\left(n_{i}+1\right)})$$

For any realization, the sum of the top *s_i _* order statistics is the maximum of the sum of all possible subsets of size *s_i _* given a family size *n_i _* + ≥ *s_i_*:

(14)
$$\sum_{r=n_{i}+1-s_{i}+1}^{n_{i}+1}X_{\mathrm{i(r)}}^{\left(n_{i}+1\right)}=\overset{max}{A\subset \left\{1,\ldots ,n_{i}+1\right\},|A|=s_{i}}\sum_{j\in A}X_{ij}$$

Applying the same principle to a cross with family size *n_i _* ≥ *s_i _*, we get

(15)
$$\sum_{r=n_{i}+1-s_{i}+1}^{n_{i}+1}X_{\mathrm{i(r)}}^{n_{i}+1}\geq \overset{max}{A\subset \left\{1,\ldots ,n_{i}\right\},|A|=s_{i}}\sum_{j\in A}X_{ij}=\sum_{r=n_{i}-s_{i}+1}^{n_{i}}X_{i(r)}^{\left(n_{i}\right)}$$

This relies on pointwise inequality: An increase in family size from *n_i _* to *n_i _* + 1 adds another draw from the distribution of the random variable to the *n_i _* realizations. This additional sample can either be larger than the smallest of the current top *s_i_* individuals, then it will replace it and increase the sum of the top *s_i_* order statistics. Or it will be smaller, then the sum of the top *s_i_* order statistics will remain the same. Hence,

(16)
$$E(Y_{i}{)}_{n_{i}}\;\leq \;E(Y_{i}{)}_{n_{i}+1}$$

It follows that *S_i_*, *R_i_* and E(*G_i_*) are non-decreasing in *n_i_* for any selection scheme selecting a fixed number of *s_i_* individuals. This monotonicity result does not rely on any assumptions about expected order statistics and should hold for continuous, non-degenerate trait distributions.

For proving concavity of E(*G_i_*), i.e. that $\frac{\partial E(G_{i})}{\partial n_{i}}$ is non-decreasing, we need to show that Equation 11 holds true. The marginal selection gain is defined:

(17)
$$\Delta_{n_{i}}:=E(Y_{i}{)}_{n_{i}+1}\;-\;E(Y_{i}{)}_{n_{i}}$$

If we draw another sample $X_{i,n_{i}+1}$ from the distribution of *X_i_*, we increase the family size from *n_i_* +1. If the genotypic value of individual $X_{i,n_{i}+1}>X_{\left(n_{i}-s_{i}+1\right)}^{\left(n_{i}\right)}$, the marginal selection gain is

(18)
$$\Delta_{n_{i}}=\frac{1}{s_{i}}E\left[\left(X_{i,n_{i}+1}-X_{\left(n_{i}-s_{i}+1\right)}^{\left(n_{i}\right)}\right)\right]$$

Since in stochastic ordering, $X_{\left(n_{i}-s_{i}+1\right)}^{\left(n_{i}\right)}\leq_{\mathrm{st}}X_{\left(n_{i}+1-s_{i}+1\right)}^{\left(n_{i}\right)}$, and E $\left[(X_{i}-x{)}_{+}\right]$ under our assumptions is decreasing in *x*, convex and continuous,

(19)
$$E[(X_{i}-X_{i(n_{i}-s_{i}+1)}^{\left(n_{i}\right)})]\geq E[(X_{i}-X_{i(n_{i}-s_{i}+1)}^{\left(n_{i}+1\right)})]$$

and

(20)
$$\Delta_{n_{i}}\geq \Delta_{n_{i}+1}.$$

Thus, the marginal selection gains are diminishing and *S_i_*, *R_i_* and E(*G_i_*) are concave in *n_i_*.
