# Supplementary material for: Optimization of family sizes in sets of crosses with a greedy allocation strategy based on automatic differentiation
Source: Front Plant Sci. 2026 Apr 30;17:1727383. doi: 10.3389/fpls.2026.1727383 (PMC13171524; doi:10.3389/fpls.2026.1727383)
Supplement: Supplementary file 1 [file DataSheet1.pdf]

Supplementary Material for Okoye et al. 2026

R Code for the optimization algorithm

Population parameters

```
# Means of the experimental dataset
mmu <- c(82.85, 94.23, 87.10, 83.53,
         85.88, 97.26, 90.13, 86.56,
         85.08, 96.45, 89.33, 85.75,
         82.95, 94.33, 87.20, 83.63,
         85.93, 97.31, 90.18, 86.61)

# Segregation variances of experimental dataset
vr  <- c( 8.73, 1.46, 5.23, 11.91,
         11.65, 1.04, 7.32, 15.17,
         12.12, 1.20, 6.36, 12.75,
          9.83, 0.96, 3.68, 10.06,
         11.74, 1.05, 7.37, 15.20)

# Heritability
h.sq <- 0.7
```

Define parameters and functions for autograd

```
library(torch)

torch_two     <- torch_tensor(2)
torch_pi      <- torch_tensor(pi)
torch_sqrt2   <- torch_sqrt(torch_two)
torch_sqrt2pi <- torch_sqrt(torch_two * torch_pi)

sum_n_i <- function(n_list) {
  sum(sapply(n_list, function(x) as.numeric(x$item())))
}

torch_beta <- function(a, b) {
  torch_exp(torch_lgamma(a) + torch_lgamma(b) - torch_lgamma(a + b))
}

torchv_pnorm <- function(x, mean, sd) {
  0.5 * (1 + torch_erf((x - mean) / (sd * sqrt(2))))
}

torchv_dnorm <- function(x, mean, sd) {
  1 / (sd * sqrt(2 * pi)) * torch_exp(-0.5 * ((x - mean) / sd)^2)
}

torchv_xf_rn <- function(x, r, n, mu, sd) {
```

```
  x <- x$unsqueeze(2)
  r <- r$unsqueeze(1)
  x_exp <- x$expand(c(x$size(1), r$size(2)))

  beta_val <- torch_beta(r, n - r + 1)
  pnorm_x <- torchv_pnorm(x_exp, mu, sd)
  dnorm_x <- torchv_dnorm(x_exp, mu, sd)

  result <- x_exp * (1 / beta_val) *
    torch_pow(pnorm_x, r - 1) *
    torch_pow(1 - pnorm_x, n - r) *
    dnorm_x

  result$transpose(1, 2)
}

torchv_trapz <- function(y, x) {
  dx <- x[2:length(x)] - x[1:(length(x) - 1)]
  y1 <- y[ , 2:length(x)]
  y2 <- y[ , 1:(length(x) - 1)]
  avg_y <- (y1 + y2) / 2
  torch_sum(avg_y * dx, dim = 2)
}
```

Function for $\mathbf{E}(G_i)$

```
torchv_EGi <- function(s_i, n_i, mu, sd, h.sq, k=5, n_points=1000) {
  lower <- mu - k * sd
  upper <- mu + k * sd
  lower_val <- as.numeric(lower$item())
  upper_val <- as.numeric(upper$item())
  x <- torch_linspace (start=lower_val, end=upper_val, steps=n_points)
  i_vec <- torch_arange(1, s_i, dtype = torch_float())
  r_vec <- n_i - s_i + i_vec
  y_mat <- torchv_xf_rn(x, r_vec, n_i, mu, sd)
  integrals <- torchv_trapz(y_mat, x)
  (torch_mean(integrals) - mu) * h.sq
}
```

Function for optimization of n_i

```
torchv_opt_ni <- function(N, s_i, mmu, ssd, hh.sq) {
  mu <- lapply(mmu, function(x) torch_tensor(x))
  sd <- lapply(ssd, function(x) torch_tensor(x))
  ss <- lapply(s_i, function(x) torch_tensor(x))
  h.sq <- torch_tensor(hh.sq)
```

```
k <- length(mmu)  # number of components
n_i <- lapply(s_i,
              function(x) torch_tensor(x,
                                       dtype=torch_float(),
                                       requires_grad=TRUE))

deltaEGi <- numeric(k)
EGi_trace <- list()     # Stores EGi values at each step
n_i_trace <- list()     # Stores n_i values at each step
gradient_trace <- list()  # to track deltaEGi values at each step

# Initial gradient computation
for (i in seq_along(n_i)) {
  EGi <- torchv_EGi(ss[[i]], n_i[[i]], mu[[i]], sd[[i]], h.sq)
  EGi$backward()
  deltaEGi[i] <- as.numeric(n_i[[i]]$grad$item())

}
gradient_trace[[length(gradient_trace) + 1]] <- deltaEGi
# Initial trace
current_EGi <- sapply(seq_along(n_i), function(i) {
  torchv_EGi(ss[[i]], n_i[[i]], mu[[i]], sd[[i]], h.sq)$item()
})
EGi_trace[[1]] <- current_EGi
n_i_trace[[1]] <- sapply(n_i,
                         function(x) as.numeric(x$item()))

# Optimization loop
while (sum_n_i(n_i) < N) {
  max_i <- which.max(deltaEGi)

  # Increment n_i[max_i] and re-evaluate
  n_i[[max_i]] <- n_i[[max_i]]$detach()$add(1)$requires_grad_()
  EGi <- torchv_EGi(ss[[max_i]], n_i[[max_i]], mu[[max_i]], sd[[max_i]], h.sq)
  EGi$backward()
  deltaEGi[max_i] <- as.numeric(n_i[[max_i]]$grad$item())

  # Record current EGi values and n_i values
  current_EGi <- sapply(seq_along(n_i), function(i) {
    torchv_EGi(ss[[i]], n_i[[i]], mu[[i]], sd[[i]], h.sq)$item()
  })
  EGi_trace[[length(EGi_trace) + 1]] <- current_EGi
  n_i_trace[[length(n_i_trace) + 1]] <- sapply(n_i,
                                               function(x) as.numeric(x$item()))
  gradient_trace[[length(gradient_trace) + 1]] <- deltaEGi
}

# Convert trace to matrix
EGi_matrix <- do.call(rbind, EGi_trace)

# === n_i allocation ===
```

```
  n_i_matrix <- do.call(rbind, n_i_trace)

  # Grad values
  #all_grad_values <- do.call(cbind, lapply(gradient_trace, unlist))

  # Return values
  invisible(list(
    allocation = as.integer(sapply(n_i, function(x) as.numeric(x$item()))),
    n_i_matrix = n_i_matrix,
    gradient_trace = gradient_trace
  ))

}
```

Get optimal family sizes for experimental dataset

```
results <- torchv_opt_ni (
  N    = 400,
  s_i  = rep(2, 20),
  mmu  = mmu ,
  ssd  = sqrt(vr/h.sq),
  hh.sq = h.sq )

results$allocation # get final allocation to families
```

Plotting gradients and allocation of n_i

```
torchv_opt_ni_delta_allocation <- function(nrow_plot = 4,
                                           ncol_plot = 5,
                                           ab_line = F){



n_families <- ncol(results$n_i_matrix)
iterations <- nrow(results$n_i_matrix)

# Compute global y-axis limits
all_grad_values <- do.call(cbind, lapply(results$gradient_trace, unlist))
all_ni_values <- results$n_i_matrix

grad_ylim <- c(floor(min(all_grad_values)), round(max(all_grad_values)))
ni_ylim <- c(floor(min(all_ni_values)), max(all_ni_values))


# Set up plotting area
par(mfrow = c(nrow_plot, ncol_plot), mar = c(2, 2, 2, 2), oma = c(5, 5, 4, 5))

for (f in 1:n_families) {
  # Data
```

```
  ni_values <- all_ni_values[, f]
  grad_values <- sapply(results$gradient_trace, function(x) x[f])
  iter <- seq_along(ni_values)

  # Check if subplot is outer edge
  is_left   <- (f %% ncol_plot == 1)
  is_right  <- (f %% ncol_plot == 0)
  is_bottom <- (f > n_families - ncol_plot)

  # Plot gradient on left y-axis
  plot(iter, grad_values, type = "l", lwd = 2, col = "coral",
       ylim = grad_ylim, axes = FALSE, xlab = "", ylab = "",
       main = paste("Family", f))
  box()
  grid(col = "grey75", lty = "dotted")
  if (is_left) axis(2, col.axis = "coral", col = "coral", las = 1)
  if (is_bottom) axis(1)

  # Overlay n_i on right y-axis
  par(new = TRUE)
  plot(iter, ni_values, type = "l", lwd = 2,
       col = "cornflowerblue",
       ylim = ni_ylim, axes = FALSE, xlab = "", ylab = "")
  if (ab_line) abline(h = 20, lty = 2, col = "gray30")
  if (is_right) axis(4, col.axis = "cornflowerblue",
                     col = "cornflowerblue", las = 1)
}

# Global labels and title
mtext("Iteration", side = 1, outer = TRUE, line = 3, cex = 1.1)
mtext(expression(Gradient ~ E(G[i])), side = 2, outer = TRUE, line = 3,
      col = "coral", cex = 1.1)
mtext(expression(n[i] ~ "allocation"), side = 4, outer = TRUE, line = 3,
      col = "cornflowerblue", cex = 1.1)
}

# For plotting, nrow_plot * ncol_plot must correspond to the
# number of crosses or be slightly higher
# In our example, the number of crosses is k = 20 = 4 * 5
torchv_opt_ni_delta_allocation(nrow_plot = 4, ncol_plot = 5, ab_line = F)
```

Computation times

Table S1 Investigated grid of scenarios tested for evaluation of the elapsed computation time for optimization of family size n_i. Each scenario was replicated 10 times and average computation times were recorded.

	Number of crosses k		
Family size n_i	20	50	100
	$N = k \cdot n_i$		
20	400	1000	2000
50	1000	2500	5000
100	2000	5000	10000

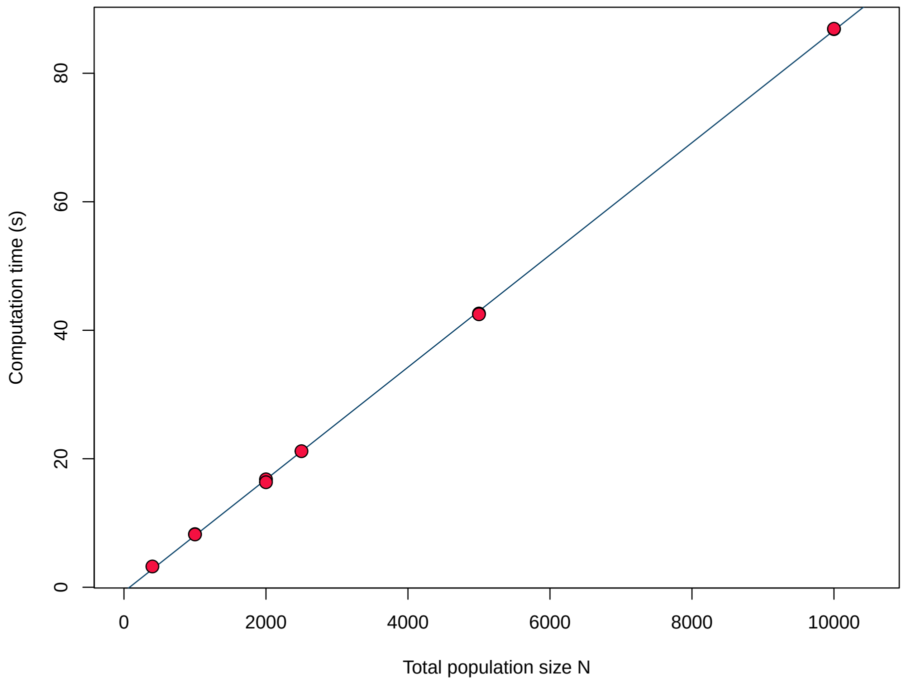

Figure S1 Elapsed computation time for the optimization of n_i in seconds based on the scenarios described in Table S1. The computation time is linear in total population size N. The effect of different $k \cdot n_i$ scenarios is negligible.

Sensitivity test for variance prediction error

Table S2 Loss in $\Delta\mathrm{E}(G)$ between variable and constant n_i in percentage due to sub-optimal progeny allocation from noisy segregation variance estimates with prediction error compared to true σ_i^{*2}. Three different levels of mean absolute prediction error were investigated for the $k = 20$ crosses of the experimental barley data set: 5 %, 10 % and 25 % of the the segregation variance σ_i^*.

	Variance prediction error	Loss in $\Delta\mathrm{E}(G)$ (%)					
Scenario	(MAVE)	$s_i = 1$	$s_i = 2$	$s_i = 3$	$s_i = 4$	$s_i = 5$	$s_i = 10$
$N = 400$, $H^2 = 0.9$	5 %	0.54	0.54	0.47	0.59	0.48	0.75
	10 %	2.20	2.16	2.06	2.18	2.05	2.28
	25 %	18.61	18.15	17.57	17.27	16.68	15.66
$N = 400$, $H^2 = 0.7$	5 %	0.51	0.53	0.43	0.51	0.43	0.63
	10 %	2.07	2.14	2.07	2.18	2.01	2.33
	25 %	23.73	21.98	20.67	20.06	19.09	17.07
$N = 1200$, $H^2 = 0.9$	5 %	0.52	0.51	0.50	0.52	0.50	0.50
	10 %	2.12	2.12	2.12	2.13	2.11	2.12
	25 %	19.57	19.06	18.70	18.45	18.24	17.49
$N = 1200$, $H^2 = 0.7$	5 %	0.54	0.52	0.51	0.52	0.50	0.53
	10 %	2.25	2.24	2.22	2.23	2.21	2.23
	25 %	21.84	21.21	20.72	20.39	20.09	19.17

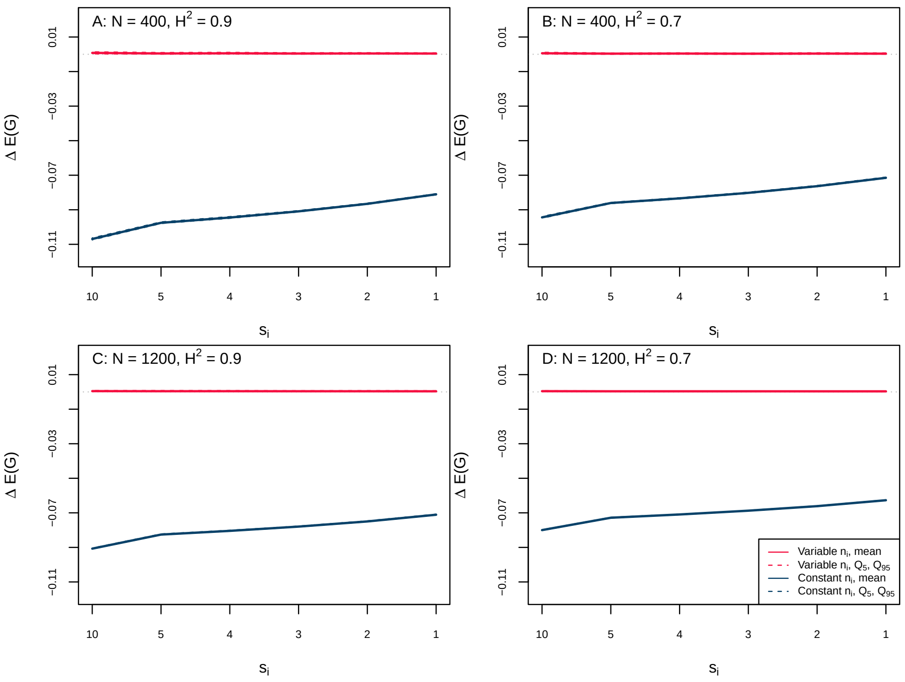

Figure S2 Results from the sensitivity test of the optimization to variance prediction error. Absolute differences $\Delta_{\text{var}} = \text{E}(G)_{\text{optimal}} - \text{E}(G)_{\text{sub-optimal}}$ (Red solid line: mean, red dotted lines: $Q_{5\%}$, $Q_{95\%}$) and $\Delta_{\text{const}} = \text{E}(G)_{\text{const}} - \text{E}(G)_{\text{sub-optimal}}$ (Blue solid line: mean, blue dotted lines: $Q_{5\%}$, $Q_{95\%}$) for simulated MAVE = 5 %. A positive sign of Δ_{var} reflects a loss in $\text{E}(G)$ compared to optimal n_i derived from "true" segregation variances σ_i^{*2} due to suboptimal allocation of n_i derived from "noisy" simulated segregation variances. A negative sign of Δ_{const} reflects a gain in $\text{E}(G)$ in compared to constant family sizes n_i.

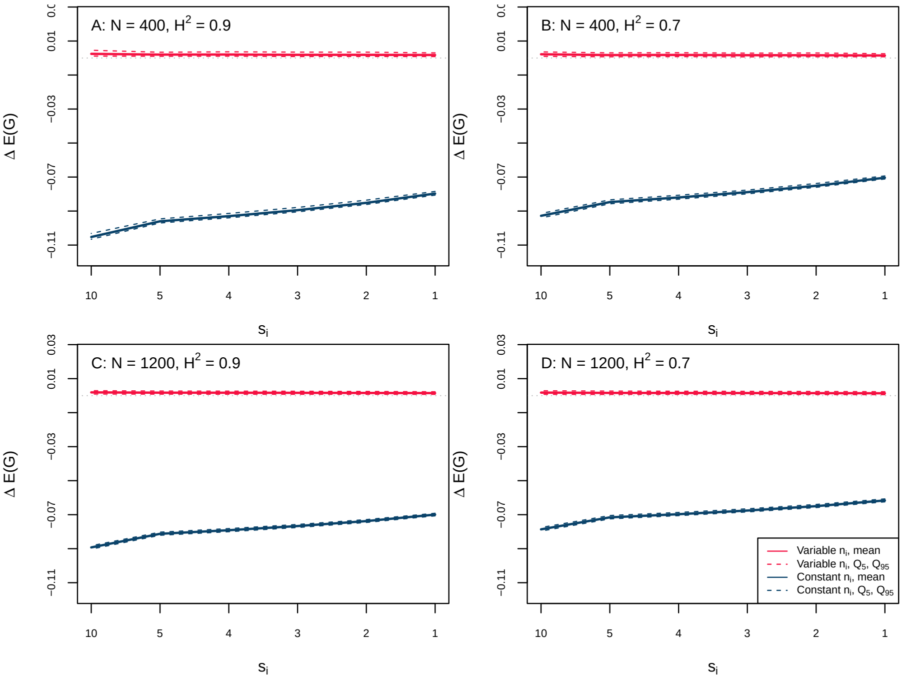

Figure S3 Results from the sensitivity test of the optimization to variance prediction error. Absolute differences $\Delta_{\text{var}} = \text{E}(G)_{\text{optimal}} - \text{E}(G)_{\text{sub-optimal}}$ (Red solid line: mean, red dotted lines: $Q_{5\%}$, $Q_{95\%}$) and $\Delta_{\text{const}} = \text{E}(G)_{\text{const}} - \text{E}(G)_{\text{sub-optimal}}$ (Blue solid line: mean, blue dotted lines: $Q_{5\%}$, $Q_{95\%}$) for simulated MAVE = 10 %. A positive sign of Δ_{var} reflects a loss in $\text{E}(G)$ compared to optimal n_i derived from "true" segregation variances σ_i^{*2} due to suboptimal allocation of n_i derived from "noisy" simulated segregation variances. A negative sign of Δ_{const} reflects a gain in $\text{E}(G)$ in compared to constant family sizes n_i.

Table S3 Optimal family sizes n_i for the $k = 20$ crosses of the experimental barley dataset as a function of σ_i^*, N and s_i. These family sizes were used to calculate $\mathrm{E}(G)$ for Figure 3 of the manuscript. The n_i for scenarios with heritability $H^2 = 0.9$ are also shown in Figure 4 of the manuscript. As the heritability serves as a scaling factor in a linear transformation for the calculation of $\mathrm{E}(G)$, it does not change the ratio of the segregation standard deviations. Consequently, the n_i are the same for both $H^2 = 0.9$ and $H^2 = 0.7$.

σ_i^*																			
2.95	1.21	2.29	3.45	3.41	1.02	2.71	3.89	3.48	1.10	2.52	3.57	3.14	0.98	1.92	3.17	3.43	1.02	2.71	3.90
$N = 400,\ H^2 = 0.9$																			
23	11	18	26	25	9	21	28	26	10	20	26	24	9	16	24	26	9	21	28
22	11	18	26	25	10	21	28	26	10	20	26	24	9	16	24	25	10	21	28
22	11	18	25	25	10	21	28	26	11	20	26	23	10	16	24	25	10	21	28
22	12	18	25	25	10	21	28	25	11	20	26	23	10	16	24	25	10	21	28
22	12	18	25	25	11	21	27	25	11	20	26	23	10	16	23	25	11	21	28
22	13	18	24	24	12	21	26	24	13	20	25	23	12	17	23	24	12	21	26
$N = 1200,\ H^2 = 0.9$																			
68	31	54	78	77	27	63	87	78	29	59	80	71	26	46	72	77	27	63	87
68	32	54	78	77	27	63	86	78	29	59	80	71	26	47	72	77	27	63	86
67	32	54	77	76	28	63	86	78	29	59	80	71	27	47	72	77	28	63	86
68	32	54	77	76	28	63	85	78	30	59	79	71	27	47	72	77	28	63	86
67	33	54	77	76	28	63	85	78	30	59	79	71	28	47	72	77	28	63	85
67	34	55	76	75	30	63	84	77	31	59	78	70	29	48	71	76	30	63	84
$N = 400,\ H^2 = 0.7$																			
23	11	18	26	25	9	21	28	26	10	20	26	24	9	16	24	26	9	21	28
22	11	18	26	25	10	21	28	26	10	20	26	24	9	16	24	25	10	21	28
22	11	18	25	25	10	21	28	26	11	20	26	23	10	16	24	25	10	21	28
22	12	18	25	25	10	21	28	25	11	20	26	23	10	16	24	25	10	21	28
22	12	18	25	25	11	21	27	25	11	20	26	23	10	16	23	25	11	21	28
22	13	18	24	24	12	21	26	24	13	20	25	23	12	17	23	24	12	21	26
$N = 1200,\ H^2 = 0.7$																			
68	31	54	78	77	27	63	87	78	29	59	80	71	26	46	72	78	27	63	86
68	32	54	77	77	27	63	86	78	29	59	80	71	27	47	72	77	27	63	86
68	32	54	77	77	28	63	85	78	29	59	80	71	27	47	72	77	28	63	85
68	32	54	77	77	28	63	85	78	30	59	79	71	27	47	72	77	28	63	85
67	33	54	77	76	28	63	85	78	30	59	79	71	28	47	72	76	29	63	85
67	34	55	76	75	30	63	84	77	31	59	78	70	29	48	71	76	30	63	84
